# A MOR Antagonist with High Potency and Antagonist Efficacy among Diastereomeric C9-Alkyl-Substituted *N*-Phenethyl-5-(3-hydroxy)phenylmorphans

**DOI:** 10.3390/molecules28145411

**Published:** 2023-07-14

**Authors:** Dana R. Chambers, Agnieszka Sulima, Dan Luo, Thomas E. Prisinzano, Arthur E. Jacobson, Kenner C. Rice

**Affiliations:** 1 Drug Design and Synthesis Section, Molecular Targets and Medications Discovery Branch, Intramural Research Program, National Institute on Drug Abuse and the National Institute on Alcohol Abuse and Alcoholism, National Institutes of Health, Department of Health and Human Services, 9800 Medical Center Drive, Bethesda, MD 20892, USA; dana.chambers92@gmail.com (D.R.C.); agnieszka.sulima@nih.gov (A.S.); 2 Department of Pharmaceutical Sciences, College of Pharmacy, University of Kentucky, 789 S. Limestone Street, Lexington, KY 40536, USA; dan.luo@uky.edu (D.L.); prisinzano@uky.edu (T.E.P.)

**Keywords:** *N*-phenethyl-5-(3-hydroxy)phenylmorphan, inhibition of forskolin-induced cAMP accumulation assay (cAMP assay)

## Abstract

The 5-(3-hydroxy)phenylmorphan structural class of compounds are unlike the classical morphinans, 4,5-epoxymorphinans, and 6,7-benzomorphans, in that they have an equatorially oriented aromatic ring rather than the axial orientation of that ring found in the classical opioids. This modified and simplified opioid-like structure has been shown to retain antinociceptive activity, depending on its stereochemistry and substituents, and some of them have been found to be much more potent than morphine. A simple C9-hydroxy-5-(3-hydroxy)phenylmorphan enantiomer was found to be about 500 times more potent than morphine in vivo. We have previously examined C9-alkenyl and hydroxyalkyl substituents in the *N*-phenethyl-5-(3-hydroxy)phenylmorphan class of compounds. Comparable C9-alkyl (methyl through butyl) substituents, with their sets of diastereomers, have not been explored. All these compounds have now been synthesized to determine the effect chain-length and stereochemistry at the C9 position in the molecule might have on their interaction with opioid receptors. We now report the synthesis and in vitro activity of 16 compounds, the C9-methyl, ethyl, propyl, and butyl diastereomers, using the inhibition of forskolin-induced cAMP accumulation assay. Several potent (sub-nanomolar and nanomolar) MOR compounds were found to be selective agonists with varying efficacy. Of greatest interest, a selective MOR antagonist was discovered; it did not display any DOR or KOR agonist activity in vitro, was three times more potent than naltrexone, and was found to antagonize the EC90 of fentanyl at MOR to a greater extent than naltrexone.

## 1. Introduction

**Figure 1:**
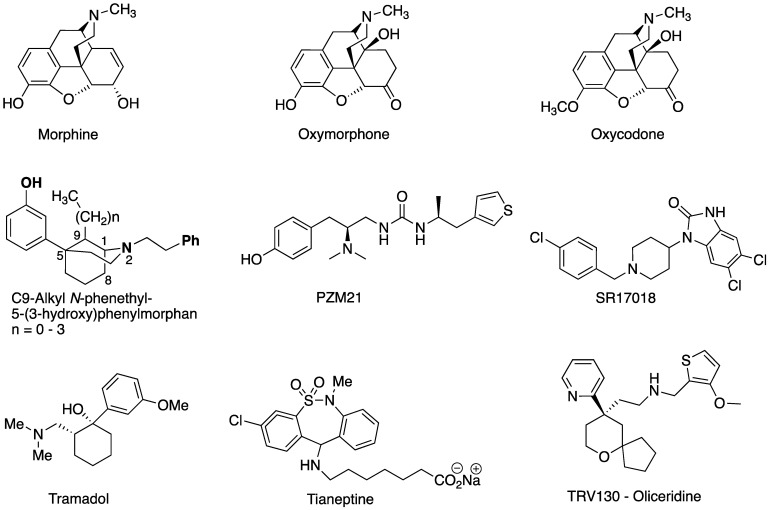
Molecular structure of potential analgesics.

The Center for Disease Control and Prevention (CDC) reported that more than 932,000 people have died since 1999 from a drug overdose. In 2020, opioids were involved in 68,630 overdose deaths (URL (accessed on 7 June 2023) https://www.cdc.gov/drugoverdose/deaths/index.html). According to the United States Department of Justice, the direct and indirect monetary cost of illicit drug use in the U.S. totaled around $193 billion in 2007. The cost in lives destroyed by the illicit use of drugs is immeasurable and, unfortunately, with increasingly potent analgesics reaching the market, those totals will only increase in future years. For these reasons, researchers have sought new types of analgesics as well as new and more selective and potent antagonists. Although no theory has succeeded in predicting the properties of a molecule that could act as a selective potent opioid antagonist, several theories have been developed that relate the properties of potential analgesics to their undesired side effects [1,2,3,4,5,6]. Some new types of analgesics (e.g., PZM21, SR17018, tramadol, tianeptine, oliceridine, Figure 1) have structures that appear to be quite different from the opioids used clinically (e.g., morphine, oxymorphone, and oxycodone (Figure 1)). Of these new compounds, only oliceridine has been approved for human use as an analgesic. It was shown to have somewhat fewer side effects than those associated with the opioids (the major opioid side effects are respiratory depression, gastrointestinal effects, tolerance, dependence, and OUD (opioid use disorder)).

The 5-(3-hydroxy)phenylmorphan molecule (Figure 1) was designed [7] as a simplified form of morphine, but that simplification introduced major changes. Morphine, and all the classical 4,5-epoxymorphinan opioids, have a rigid aromatic phenyl ring axially oriented towards the piperidine ring, unlike the 5-phenylmorphans where the phenyl ring is detached from morphine’s B-ring and equatorially oriented towards the piperidine ring. The phenyl ring in the 5-phenylmorphans can rotate around a C-C bond, unlike the rigid phenyl ring in morphine. We have employed an *N*-phenethyl substituent, rather than *N*-methyl substituent, since it has been found to increase the potency of the molecule in the chiral C9-alkyl series; although in a racemic 5-phenylmorphan, it was initially found to decrease antinociceptive activity in mice [8].

Many other types of N-substituents have been noted in the literature [9,10]. We have used the *N*-phenethyl substituent for all of our C9-diastereomers, and we maintain that, initially, as a constant for comparison of their activity in vitro. In earlier work with the 5-phenylmorphans, we determined the effect of the enantiomers of a C9-hydroxyl group and a C9-methyl group in a few chiral *N*-phenethyl-substituted 5-phenylmorphans [11]. The 1*R*,5*R*,9*S*-OH compound and the 1*R*,5*S*,9*R*-methyl compound were found to have sub-nanomolar MOR affinity in a receptor binding assay. A later study with a 1*S*,5*R*,9*R*-propyl group at C9 in the phenylmorphans was also intriguing [12]. In an inhibition of forskolin-induced cAMP accumulation assay (cAMP assay), it acted as a partial agonist (EC_50_ (potency) = 1.42 nM; %E_max_ (efficacy) = 45%). Further testing in vivo has indicated that it was unlike morphine in its locomotor effects [13]. The C9-propyl compound appeared to have some antinociceptive activity, depending on the animal species used and the pain stimulus [13]. With our previous discovery that a chiral C9-methyl [11] and the C9-propyl [12] compound had interesting activity, we thought we should design compounds that would explore the complete 3-dimensional space around the C9 area of the 5-phenylmorphan, while maintaining the *N*-phenethyl substituent as a constant. A C9-substituent introduced a third chiral atom in the molecule. However, with one chiral atom (at C5) fixed, we would only need four diastereomers for every C9-substituent. To examine C9-alkyl substituents from methyl through butyl, we synthesized a total of 16 compounds. Three of these (two C9-methyl compounds and the C9-propyl) had previously been prepared [11,12], and they were reexamined in vitro for comparison with the novel compounds. We have, in this same manner, examined C9-alkenyl compounds [14] and hydroxyalkyl compounds and found interesting MOR partial agonists and potent MOR antagonists among them. The agonists and antagonists were distinguishable using molecular modeling [14]. The antagonists were found to interact with the inactive (4DKL) MOR crystal structures and agonists were found to interact with the active (6DDF) MOR crystal structures [14]. The determination that several C9-alkenyl compounds were of interest [15] gave us hope that we would find interesting alkyl compounds. 

## 2. Results and Discussion

### 2.1. Synthesis of Diastereomeric C9-Alkyl-5-phenylmorphans 

**Scheme 1:**
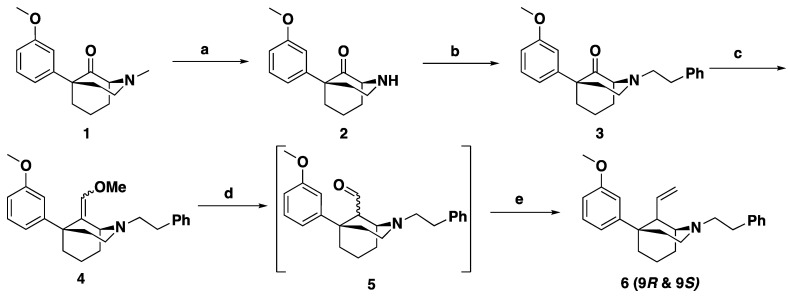
General route for the synthesis of the 9*R* and 9*S*-alkenyl intermediate (**6**). Reagents and conditions: (**a**) 1. CNBr, K_2_CO_3_, MeCN, reflux 4 h, 2. 3 N aq HCl, MeOH, reflux 16 h; (**b**) Ph(CH_2_)_2_Br, K_2_CO_3_, MeCN, reflux 16 h; (**c**) LiHMDS (methoxymethyl)triphenylphosphonium chloride, THF, 0 °C; (**d**) 6 N aq HCl; (**e**) Wittig conditions.

The synthesis of the C9 ethyl though butyl series (*n* = 1–3, Figure 1) of phenylmorphans was achieved through the route shown in Scheme 1. The racemic *N*-methyl compound **1** (Scheme 1) was synthesized from commercially available compounds following literature procedures [7,16]. Using the procedure of Hiebel et al. [11], optical resolution to separate the enantiomers of ±**1** was achieved using tartaric acid, and the enantiomers were purified to >99% ee (as determined using ^1^H NMR). The tartrate salt of (1*S*,5*S*)-9-ketophenylmorpohan (1*S*, 5*S*-**1**) is shown in Scheme 1. For each enantiomer, the *N*-methyl substituent was replaced with an *N*-phenethyl moiety. Treatment of *N*-methyl compound **1** was with cyanogen bromide and subsequent hydrolysis with 3 N HCl yielded secondary amine **2** in high yield and purity. Further purification was unnecessary before alkylation. Treatment of secondary amine **2** with phenethyl bromide gave the alkylation product **3**. Formation of alkylated product **3** gave a >60% yield over two steps. This procedure was scalable, and reactions were run on 1 g to 26 g scale without loss of yield. 

**Scheme 2:**
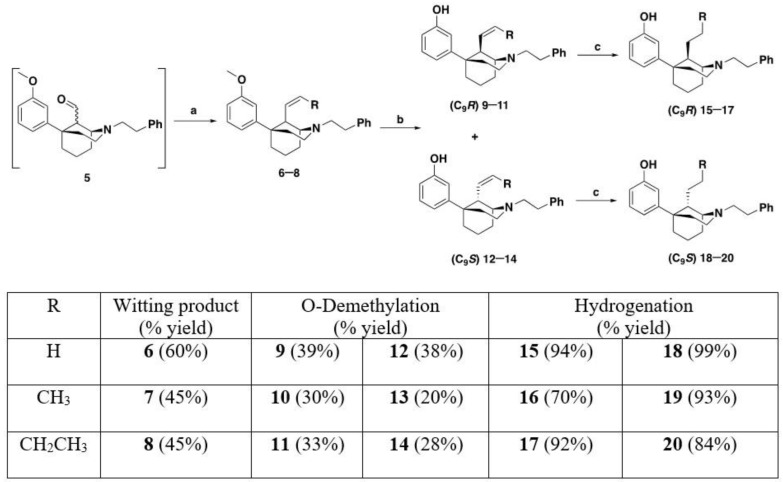
Formation and separation of C9*R* (**15**–**17**) and C9*S* (**18**–**20**) epimers. Reagents and conditions: (**a**) Wittig conditions; (**b**) BBr_3_, CH_2_Cl_2_, 0 °C–rt 4 h; (**c**) Pd/C, H_2_, HCl, 16 h.

Functionalization of the ketone at the C9 position via the formation of enol ether **4** (Scheme 1) was achieved by a Wittig reaction of **3** with methoxymethyl triphenylphosphonium chloride and LiHMDS. Installation of the C9 stereocenter was accomplished by the hydrolysis of enol ether **4** with aqueous HCl. The concentration of HCl, reaction time, and quenching conditions affect the C9 *R*:*S* ratio, as noted previously [14]. Aldehyde **5** was used immediately upon formation without purification because it was unstable to silica gel column chromatography and not stable at room temperature for longer than a few hours. From the common intermediate **5**, Wittig reactions using varying phosphonium salts (Scheme 2) were performed to obtain alkenes at C9 [14]. The Wittig reactions resulted in a mixture of epimers at the C9 position, which were not separable by silica gel column chromatography. The mixture of epimers was subject to *O*-demethylation conditions using BBr_3_ to form phenols **9**–**14** in 60–80% yields (Scheme 2). The phenol compounds **9**–**14** could be separated into their C9*S* and C9*R* diastereomers by column chromatography. 

Alkenes **9**–**14** were hydrogenated in the presence of Pd/C (0.1 equiv) to give the desired alkyl compounds **15**–**20** (Scheme 2) in the 1*S*,5*R* series. The use of HCl salts of **9**–**14**, or the treatment of the free base with one equivalent of HCl, for the hydrogenation reactions was necessary for high yielding reductions. When HCl was not employed, reaction yields dropped significantly. Alkyl compounds **15**–**20** were purified by column chromatography and isolated as oils. All the free base oils were treated with HCl in isopropanol to form the HCl salts as white solids, except for the C9 butyl compound **20**, which was isolated as the tartrate salt.

**Scheme 3:**
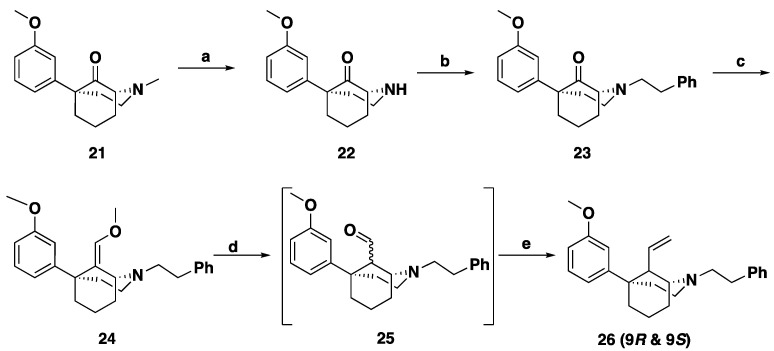
Synthesis of 1*R*,5*S* enantiomers (**26**, 9*R* & 9*S*). Reagents and conditions: (**a**) 1. CNBr, K_2_CO_3_, MeCN, reflux h, 2. 3 N aq HCl, MeOH, reflux 16 h; (**b**) Ph(CH_2_)_2_Br, K_2_CO_3_, MeCN, relux 16 h; (**c**) LiHMDS (methoxymethyl)triphenylphosphonium chloride, THF, 0 °C; (**d**) 6 N aq HCl; (**e**) Wittig conditions.

The enantiomers (**21**–**26**) of compounds **15**–**20** were synthesized from the tartrate salt of (1*R*,5*S*)-5-(3-methoxyphenyl)-2-methyl-2-azabicyclo[3.3.1]nonan-9-one (1*R*,5*S*–**21**, Scheme 3) by the same synthetic steps as in Scheme 1.

**Scheme 4:**
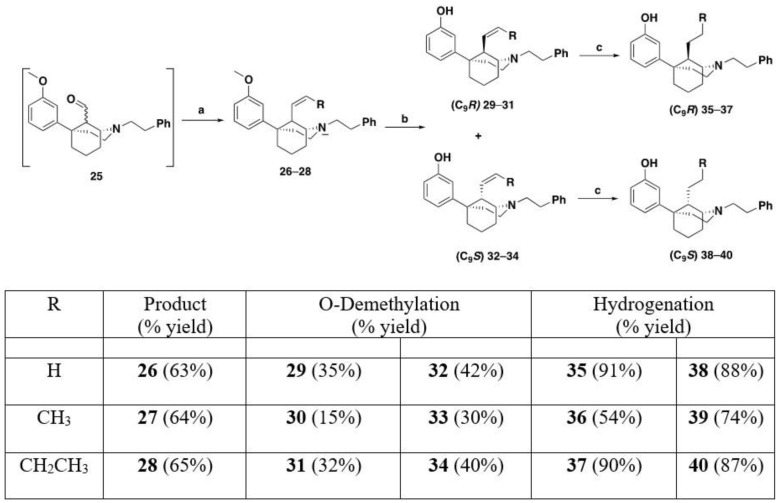
Synthesis of C9*R* (**35**–**37**) and C9*S* (**38**–**40**) epimers. Reagents and conditions: (**a**) Wittig conditions; (**b**) BBr_3_, CH_2_Cl_2_, 0 °C–rt 4 h; (**c**) H_2_, Pd/C, MeOH, 16 h.

Using the same procedures described in Scheme 2, the Wittig reactions with aldehyde **25** (Scheme 4) yielded compounds **26**–**28** as a mixture of epimers at the C9 position that were not easily separable. *O*-Demethylation of **26**–**28** with BBr_3_ gave phenols **29**–**34**, respectively. The phenolic compounds were separable by silica gel column chromatography and provided C9*R* and C9*S* compounds. Hydrogenation using Pd/C gave the final products **35**–**40**.

**Scheme 5:**
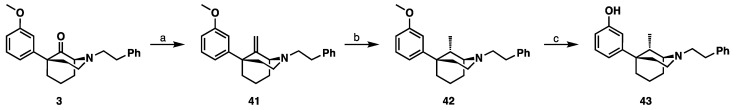
Resynthesis of 9*S*-methyl-1*S*,5*R*-5-phenylmorphan (**43**) [11]. Reagents and conditions: (**a**) Tebbe’s reagent, THF, 3 h; (**b**) H_2_, Pd/C, MeOH, quant; (**c**) BBr_3_, CH_2_Cl_2_, 4 h.

The C9*S*-methyl compound (**43**) in the 1*S*,5*R*-series were synthesized (Scheme 5) from compound **3** using the literature procedure [11]. The alkylation product **3** olefination using Tebbe’s reagent gave alkene **41** in a 71% yield (Scheme 5). Reduction of **41** gave stereo-selectively the C9*S*-methyl **42** with only a small impurity of the C9*R*-methyl diastereomer. The final *O*-demethylated product **43** [11] was achieved by treating **42** with BBr_3_.

**Scheme 6:**
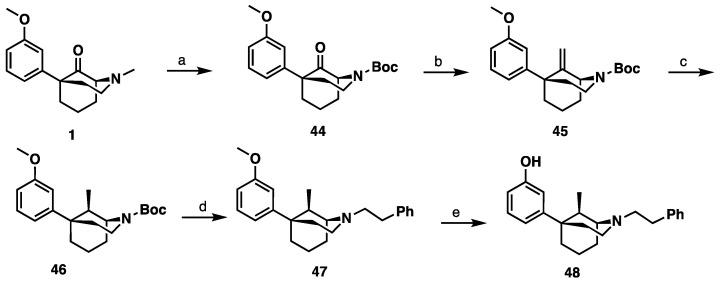
Synthesis of 9*R*-methyl-1*S*,5*R*-5-phenylmorphan (**48**). Reagents and conditions: (**a**) 1. BrCN, K_2_CO_3_, CH_3_CN, reflux, 2 h; 2. 3 M HCl, MeOH, reflux, 16 h; 3. Boc_2_O, Et_3_N, DMAP, MeOH, 1 h; (**b**) Tebbe’s reagent, THF, 16 h; (**c**) H_2_, Pd/C, MeOH; (**d**) 1. TFA, CH_2_Cl_2_, 1.5 h; 2. Phenethyl bromide, K_2_CO_3_, CH_3_CN, 16 h. (**e**) BBr_3_, CH_2_Cl_2_, 4 h.

The C9*R*-methyl diastereomer (**48**, Scheme 6) had not been previously made as the literature method [11] selectively formed only the C9*S*-methyl epimer **43**. To obtain the C9*R*-epimer, *N*-methyl phenylmorphan **1** (Scheme 6) was subjected to *N*-demethylation conditions followed by a Boc protection resulting in **44**. Using a Tebbe’s olefination reaction to form the C9 methylene compound **45**, followed by a hydrogenation reaction, resulted in a mixture of the desired C9*R*-stereochemistry in **46** and the C9*S*-epimer. Isolation and subsequent deprotection of the C9*R* isomer and the introduction of the phenethyl moiety resulted in compound **47**. Finally, *O*-demethylation using standard conditions yielded the desired C9*S*-methyl compound **48**. 

The selectivity of hydrogenation could be shifted based on the substituent on the oxygen and nitrogen (Table 1). With a phenethyl group on nitrogen and a *m*-methoxy substituted aromatic ring, the C9*S* epimer is formed exclusively. After *O*-demethylation the same hydrogenation conditions result in a 5:1 ratio still favoring the C9*S* epimer. Alternatively, when a Boc group is exchanged for the phenethyl group on the nitrogen and a *meta*-methoxoyphenyl moiety is present the selectivity shifts to favor the C9*R* epimer in a 3:1 ratio. This observation was exploited to obtain all four diastereomers of the C9-methyl phenylmorphan (e.g., Scheme 5 for the C9*R*-methyl in the 1*S*,5*R* series using the starting material in reaction #3 in Table 1; the C9*S* epimer was obtained using the starting material in reaction 1 in Table 1). 

**Scheme 7:**
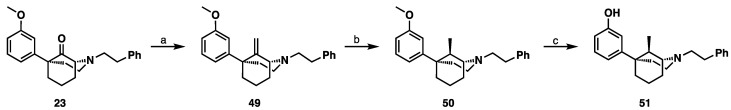
Synthesis of 9*R*-methyl-1*R*,5*S*-5-phenylmorphan (**51**). Reagents and conditions: (**a**) Tebbe’s reagent, THF, 3 h; (**b**) H_2_, Pd/C, MeOH, quantitative; (**c**) BBr_3_, CH_2_Cl_2_, 4 h.

**Scheme 8:**
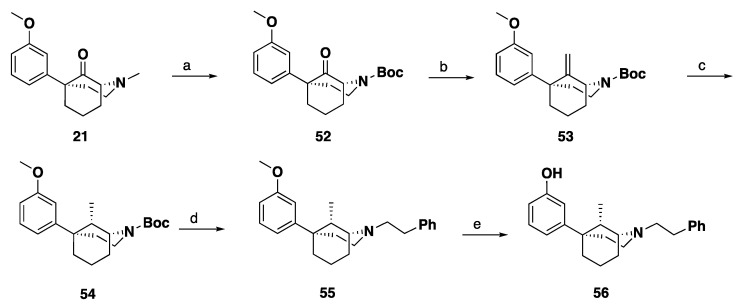
Synthesis of 9*S*-methyl-1*R*,5*S*-5-phenylmorphan (**56**). Reagents and conditions: (**a**) 1. Cyanogen bromide, K_2_CO_3_, CH_3_CN, HCl, 16 h; 2. Et_3_N, 4-dimethylaminopyridine, di-tert-butyl decarbonate, MeOH, 1 h; (**b**) Tebbe’s reagent, THF, 16 h; (**c**) H_2_, Pd/C, MeOH; (**d**) 1. TFA, CH_2_Cl_2_, 1.5 h; 2. Phenethyl bromide, K_2_CO_3_, CH_3_CN, 16 h. (**e**) BBr_3_, CH_2_Cl_2_, 4 h.

Using the same procedures described in Scheme 5 and Scheme 6, the two C9-methyl diastereomers were obtained for the 1*R*,5*S* series (Scheme 7 and Scheme 8). The C9S and C9*R*-methyl compounds were differentiated based on the previously determined X-ray diffraction analyses of **51** [11].

### 2.2. Forskolin-Induced cAMP Accumulation Assay for In Vitro Determination of the Potency and Efficacy of the Diastereomers 

Compounds were examined for their functional activity using the inhibition of forskolin-induced cAMP accumulation assay (Table 2). Four diastereomers for each of the C9-alkyl compounds, C9-methyl, ethyl, propyl, and butyl were evaluated. All except three of these (compounds **43** [11], **51** [11] and **16** [12]) in Table 2 are novel, and the three formerly known compounds have been included and re-evaluated so that the data on the complete series could be compared.

The C9-methyl series were of most interest in that one of them, **48**, was a selective potent MOR antagonist (IC_50_ = 3.9 nM) with a high %I_max_ = 153%. It did not have DOR or KOR agonist activity, unlike naltrexone, which acts as a potent partial kappa agonist (KOR EC_50_ = 0.6 nM). The C9-methyl antagonist **48** was also found to antagonize the EC90 of fentanyl at MOR to a greater extent than naltrexone (%I_max_ = 153% vs 104% for naltrexone). 

The enantiomer of **48**, compound **56**, was found to be a potent sub-nanomolar partial agonist (EC_50_ = 0.8 nM, %E_max_ = 39%), and the diastereomer of **56**, compound **51**, was a potent full agonist (EC_50_ = 1.89 nM, %E_max_ = 95.7%). As agonists, compounds **56** and **51** were found to be highly selective at MOR (EC_50_ > 10,000 nM for DOR and KOR). Whereas the reference MOR agonist morphine was less potent (EC_50_ = 5.8 nM) than the three compounds **48**, **56**, and **51**, and was a partial agonist at DOR (EC_50_ = 525 nM, E_max_ = 75%) and KOR (EC_50_ = 346 nM, E_max_ = 86%). The remaining, previously described diastereomer **43** was the least potent partial C9-methyl agonist (EC_50_ = 16.1 nM, %E_max_ = 78%). These data exemplified our findings with diastereomers at C9 in the 5-phenylmorphan series and could have very different properties in vitro, and their activity was not predictable in silico. We previously determined that agonists and antagonist C9-substituted phenylmorphans could be distinguished using molecular modeling [14], but that partial and full agonists could not be differentiated.

The C9-ethyl diastereomers were all potent agonists: one set of these isomers, **15** and **38** were potent partial agonists ((EC_50_ = 1.4 nM and 4.9 nM), with weak efficacy (%E_max_ = 48% and 31%, respectively), and the other set of enantiomers, **18** and **35**, were potent full agonists (EC_50_ = 3.4 nM and 1.0 nM, %E_max_ = 97% and 96%), respectively. Compound **35** was also found to be a low efficacy DOR partial agonist and to have weak antagonist activity at KOR. With the C9-propyl diastereomers, only one compound was a potent full agonist, **36**. It had nanomolar potency (EC_50_ = 3.2 nM, %E_max_ = 94%). The other three C9-propyl compounds were partial agonists, compound **16** with very high potency and low efficacy (EC_50_ = 1.4 nM, %E_max_ = 46%), and **19** and **39** with lower potency (EC_50_ = 36 nM and 7.3 nM, respectively). The latter compound, **39**, had very little efficacy (%E_max_ = 24%). The C9-butyl compounds **20**, **37**, and **40**, were relatively inactive (EC_50_ = 53 (**37**) to 880 nM (**20**)), and only **17** had some potency (EC_50_ = 7.2 nM), as a partial agonist, but it had little efficacy (%E_max_ = 32%).

## 3. Materials and Methods

### 3.1. General Information

Melting points were determined on a Mettler Toledo MP70 and are uncorrected. Proton and carbon nuclear magnetic resonance (^1^H and ^13^C NMR) spectra (Figures S1–S12 in Supplementary Material) were recorded on a Varian Gemini-400 spectrometer in CDCl_3_ (unless otherwise noted) with the values given in ppm (TMS as internal standard) and J (Hz) assignments of ^1^H resonance coupling. The analyses were performed on the free base, unless otherwise noted. Mass spectra (HRMS) were recorded on a Waters (Mitford, MA, USA) Xevo-G X5 QTof. The optical rotation data were obtained on a PerkinElmer polarimeter model 341. Thin layer chromatography (TLC) analyses were carried out on Analtech silica gel GHLF 0.25 mm plates using various gradients of CHCl_3_/MeOH containing 1% NH_4_OH or gradients of EtOAc/*n*-hexane. Visualization was accomplished under UV light or by staining in an iodine chamber. Flash column chromatography was performed with Fluka silica gel 60 (mesh 220–400). Flash column chromatography was performed using RediSep Rf normal phase silica gel cartridges. Robertson Microlit Laboratories, Ledgewood, N.J., performed elemental analyses, and the results were within ±0.4% of the theoretical values. Experimental procedures for intermediates, and their characterization, can be found in a previous publication [14]. Compounds **16**, **43**, and **51** in Table 2 were discussed in previous publications [11,12].

### 3.2. Synthesis

General procedure for hydrogenation of C9-alkene to C9-alkane. The HCl salt of the alkene was dissolved in MeOH (10 mL) and was transferred to a Parr shaker degassed with argon. The solution was treated with palladium on carbon (Escat 103), and the Parr shaker was pressurized to 20 psi with H_2_ and was shaken at 23 °C for 16 h. Upon completion, the reaction mixture was filtered through Celite to remove the palladium and was washed several times with methanol. The filtrate was washed with saturated NaHCO_3_, dried, and concentrated in vacuo. The resulting residue was purified by silica gel column chromatography 0–60% EtOAc: Hexanes. 

3-((1*S*,5*R*,9*R*)-9-Ethyl-2-phenethyl-2-azabicyclo[3.3.1]nonan-5-yl)phenol (**15**). The HCl salt of alkene **9** (145 mg, 1 equiv, 378 µmol) was hydrogenated according to the general procedure using palladium on carbon (Escat 103) (4.02 mg, 0.1 equiv, 37.8 µmol) to give a white foam (125 mg, 95% yield). The HCl salt of **15** was formed in *i*PrOH with 37% HCl (0.1 mL) and recrystallized from ethanol to give a white solid: mp 247–250 °C. ^1^H NMR (400 MHz; CD_3_OD): δ 7.39–7.33 (m, 4H), 7.32–7.26 (m, 1H), 7.18–7.15 (m, 1H), 6.80 (t, *J* = 5.6 Hz, 1H), 6.76 (s, 1H), 6.65 (dd, *J* = 8.0, 2.0 Hz, 1H), 3.93–3.92 (m, 1H), 3.73–3.51 (m, 3H), 3.44–3.35 (m, 1H), 3.25–3.17 (m, 1H), 3.06–2.98 (m, 1H), 2.51–2.39 (m, 2H), 2.31–2.21 (m, 2H), 2.11–1.99 (m, 3H), 1.90–1.82 (m, 2H), 1.48–1.40 (m, 1H), 1.29–1.23 (m, 1H), 1.19 (d, *J* = 7.0 Hz), and 0.90 (t, *J* = 7.4 Hz, 3H). ^13^C NMR (101 MHz; CD_3_OD): δ 158.8, 150.2, 137.5, 130.6, 130.0, 129.9, 128.4, 117.3, 114.2, 113.3, 56.5, 55.6, 51.7, 46.7, 42.0, 39.0, 31.5, 28.9, 25.1, 22.1, 19.2, and 12.0; HRMS-ESI (*m*/*z*): [M + H^+^] calcd for: C_24_H_32_NO 350.2484; found: 350.2487; Anal. Calcd. For C_24_H_32_ClNO·0.65 H_2_O: C, 72.49%; H, 8.44%; and N, 3.52%. Found C, 72.54%; H, 8.64%; N, 3.58%; ${\left[\alpha \right]}_{20}^{D}$ 18.6° (c 0.4, CHCl_3_).

3-((1*S*,5*R*,9*R*)-9-Butyl-2-phenethyl-2-azabicyclo[3.3.1]nonan-5-yl)phenol (**17**). The HCl salt of alkene **11** (273 mg,1 equiv, 663 µmol) was was hydrogenated according to the general procedure using palladium on carbon (Escat 103) (4.02 mg, 0.1 equiv, 37.8 µmol) to afford **17** as a white foam (230 mg, 92% yield). The HCl salt of **17** was formed in *i*PrOH with 37% HCl (0.1 mL) and recrystallized from ethanol to give a white solid: mp 257–260 °C. ^1^H NMR (400 MHz; CD_3_OD): δ 7.39–7.34 (m, 4H), 7.32–7.27 (m, 1H), 7.19–7.15 (m, 1H), 6.81 (d, *J* = 8.0 Hz, 1H), 6.76 (s, 1H), 6.66 (dd, *J* = 8.0, 1.9 Hz, 1H), 3.88–3.86 (m, 1H), 3.73–3.65 (m, 1H), 3.57 (ddd, *J* = 18.9, 12.7, 6.4 Hz, 2H), 3.45–3.38 (m, 1H), 3.20 (dddd, *J* = 11.4, 6.1, 6.0, 5.4 Hz, 1H), 3.05–2.98 (m, 1H), 2.48–2.34 (m, 3H), 2.26–2.21 (m, 1H), 2.07–1.99 (m, 3H), 1.89–1.81 (m, 2H), 1.46–1.10 (m, 6H), 0.85 (t, *J* = 7.2 Hz, 3H). ^13^C NMR (101 MHz; CD_3_OD): δ 158.8, 150.2, 137.5, 130.6, 130.0, 129.9, 128.4, 117.3, 114.2, 113.4, 56.4, 56.4, 51.7, 44.9, 41.8, 38.9, 31.5, 30.7, 28.9, 26.1, 25.1, 23.5, 22.1, and 14.3. HRMS-ESI (*m*/*z)*: [M + H^+^] calcd for: C_26_H_36_NO 378.2797; found 378.2796; C_26_H_36_ClNO·0.15 H_2_O calc. C: 74.94%; H: 8.78%; N: 3.36%; found C: 74.87%; H: 8.75%; N: 3.31%; and ${\left[\alpha \right]}_{20}^{D}$ 23.4° (c 0.46, CHCl_3_).

3-((1*S*,5*R*,9*S*)-9-Ethyl-2-phenethyl-2-azabicyclo[3.3.1]nonan-5-yl)phenol (**18**). The HCl salt of alkene **12** (276 mg,1 equiv, 719 µmol) was dissolved in MeOH (15 mL) and was hydrogenated according to the general procedure using palladium on carbon (Escat 103) (7.65 mg, 0.1 equiv, 71.9 µmol) to afford **18** as a white foam (249 mg, 99% yield). The HCl salt of **18** was formed in *i*PrOH with 37% HCl (0.1 mL) and recrystallized from ethanol to give a white solid: mp 249–253 °C. ^1^H NMR (400 MHz; CD_3_OD): δ 7.39–7.34 (m, 4H), 7.32–7.26 (m, 1H), 7.17 (t, *J* = 8.0 Hz, 1H), 6.87 (d, *J* = 7.8 Hz, 1H), 6.85–6.81 (m, 1H), 6.66 (dd, *J* = 8.0, 2.2 Hz, 1H), 3.81 (s, 1H), 3.71–3.58 (m, 2H), 3.58–3.46 (m, 2H), 3.19–3.11 (m, 2H), 2.40–2.32 (m, 2H), 2.18–1.89 (m, 7H), 1.40–1.31 (m, 1H), 1.22–1.14 (m, 1H), and 0.86 (t, *J* = 7.5 Hz, 3H). ^13^C NMR (100 MHz; CD_3_OD): δ 158.8, 149.9, 137.8, 130.6, 130.02, 129.89, 128.3, 117.6, 114.3, 113.7, 59.1, 57.2, 51.1, 46.5, 39.6, 38.6, 31.9, 28.8, 20.8, 20.5, 17.3, and 12.0; HRMS-ESI (*m*/*z*): [M + H^+^] calcd for: C_24_H_32_NO 350.2484; found: 350.2487; Anal. Calcd. For C_24_H_32_ClNO·0.2 H_2_O: C, 73.99%; H, 8.38%; N, 3.60%. Found C, 73.61%; H, 7.98%; N, 3.51%; ${\left[\alpha \right]}_{20}^{D}$ −22.2° (c 1.20, CHCl_3_). 

3-((1*S*,5*R*,9*S*)-2-Phenethyl-9-propyl-2-azabicyclo[3.3.1]nonan-5-yl)phenol (**19**). The HCl salt of alkene **13** (425 mg,1 equiv, 1.07 mmol) was dissolved in MeOH (15 mL) and was hydrogenated according to the general procedure using palladium on carbon (Escat 103) (11.4 mg, 0.1 equiv, 107 µmol) to afford **19** as a white foam (360 mg, 93% yield). The HCl salt of **19** was formed in *i*PrOH (1 mL) with 37% HCl (0.1 mL) and recrystallized from hot ethanol (4 mL) to give a white solid: mp 240–242 °C; ^1^H NMR (400 MHz; CD_3_OD): δ 7.37–7.33 (m, 4H), 7.28 (dt, *J* = 8.6, 4.3 Hz, 1H), 7.17 (t, *J* = 7.9 Hz, 1H), 6.89–6.84 (m, 2H), 6.66 (dd, *J* = 8.0, 2.0 Hz, 1H), 3.76 (s, 1H), 3.65–3.58 (m, 2H), 3.54–3.49 (m, 2H), 3.20–3.13 (m, 2H), 2.55 (d, *J* = 10.2 Hz, 1H), 2.42–2.35 (m, 1H), 2.18–1.91 (m, 8H), 1.40–1.30 (m, 2H), 1.20–1.00 (m, 2H), 0.83 (t, *J* = 7.1 Hz, 3H). ^13^C NMR (100 MHz; CD_3_OD): δ 158.7, 149.9, 137.8, 130.5, 129.99, 129.91, 128.3, 117.7, 114.3, 113.8, 59.4, 57.2, 51.1, 44.1, 39.5, 38.5, 31.8, 29.8, 28.8, 21.2, 20.9, 17.3, 14.3. HRMS-ESI (*m*/*z*): [M + H^+^] calcd for: C_25_H_34_NO 364.2640; found 364.2635; Anal. Calcd. For C_25_H_34_ClNO·0.75 H_2_O·0.05 C_2_H_6_O: C: 72.5%; H: 8.68%; N: 3.37%; found C: 72.54%; H: 8.63%; N: 3.39%; ${\left[\alpha \right]}_{20}^{D}$ −31.0° (c 0.59, CHCl_3_).

3-((1*S*,5*R*,9*S*)-9-Butyl-2-phenethyl-2-azabicyclo[3.3.1]nonan-5-yl)phenol (**20**). The HCl salt of alkene **14** (324 mg,1 equiv, 786 µmol) was hydrogenated according to the general procedure using palladium on carbon (Escat 103) (8.37 mg, 0.1 equiv, 78.6 µmol) to afford **20** as a white foam (250 mg, 84% yield). The tartrate salt of **20** was formed in *i*PrOH with D-tartaric acid and recrystallized from hot ethanol to give a white solid: mp 193–196 °C. ^1^H NMR (400 MHz; CD_3_OD): δ 7.35–7.32 (m, 4H), 7.26 (ddd, *J* = 8.4, 5.7, 2.9 Hz, 1H), 7.15 (dd, *J* = 10.4, 6.0 Hz, 1H), 6.87 (d, *J* = 7.0 Hz, 2H), 6.66–6.64 (m, 1H), 4.47 (s, 2H), 3.75 (s, 1H), 3.63–3.58 (m, 2H), 3.48 (dd, *J* = 10.5, 6.2 Hz, 2H), 3.18–3.11 (m, 2H), 2.52–2.37 (m, 2H), 2.18–2.01 (m, 4H), 2.00–1.88 (m, 3H), 1.35–1.03 (m, 7H), 0.80 (t, *J* = 6.9 Hz, 3H). ^13^C NMR (101 MHz; CD_3_OD): δ 177.1, 158.7, 150.2, 138.1, 130.5, 130.0, 129.9, 128.2, 117.8, 114.3, 113.8, 74.3, 59.3, 57.3, 50.6, 43.8, 39.2, 38.5, 31.8, 30.3, 28.9, 27.2, 23.6, 20.8, 17.7, and 14.2; HRMS-ESI (*m*/*z*): [M + H^+^] calcd for: C_26_H_36_NO 378.2797; found 378.2799; Anal. Calcd. For C_25_H_32_ClNO ·0.1 H_2_O: C, 75.11%; H, 8.12%; N, 3.5%. Found C, 75.00%; H, 7.87%; N, 3.42%; C_30_H_41_NO_7_·0.2 H_2_O C: 67.83; H: 7.85; N: 2.64; found C: 67.62; H: 7.63; N: 2.68; and ${\left[\alpha \right]}_{20}^{D}$ −28.4° (c 0.40, CHCl_3_).

3-((1*R*,5*S*,9*R*)-9-Ethyl-2-phenethyl-2-azabicyclo[3.3.1]nonan-5-yl)phenol (**35**). The HCl salt of alkene **29** (300 mg,1 equiv, 781 µmol) was dissolved in MeOH (15 mL) and was hydrogenated according to the general procedure using palladium on carbon (Escat 103) (8.31 mg, 0.1 equiv, 78.1 µmol) to afford **36** and isolated as a white foam (250 mg, 91% yield). The HCl salt of **36** was formed in *i*PrOH with 37% HCl (0.1 mL) and recrystallized from ethanol to give a white solid: mp 231–235°; ^1^H NMR (400 MHz; CD_3_OD): δ 7.35–7.31 (m, 4H), 7.26 (dq, *J* = 8.5, 4.3 Hz, 1H), 7.17–7.13 (m, 1H), 6.86–6.82 (m, 2H), 6.64 (dd, *J* = 8.0, 1.8 Hz, 1H), 3.78–3.76 (m, 1H), 3.65–3.57 (m, 2H), 3.53–3.46 (m, 2H), 3.19–3.08 (m, 2H), 2.42–2.31 (m, 2H), 2.17–1.85 (m, 7H), 1.37–1.27 (m, 1H), 1.20–1.10 (m, 1H), 0.84 (t, *J* = 7.4 Hz, 3H); ^13^C NMR (101 MHz; CD_3_OD): δ 158.8, 150.0, 137.8, 130.6, 129.99, 129.91, 128.3, 117.6, 114.3, 113.7, 59.0, 57.2, 51.1, 46.4, 39.5, 38.6, 31.8, 28.8, 20.8, 20.5, 17.3, and 12.0; HRMS-ESI (*m*/*z*): [M + H^+^] calcd for: C_24_H_32_NO 350.2484; found: 350.2484; Anal. Calcd. For C_24_H_32_ClNO: C, 74.68%; H, 8.36%; N, 3.63%. Found C, 74.49%; H, 7.98%; N, 3.59%; ${\left[\alpha \right]}_{20}^{D}$ 20.4° (c 0.80, CHCl_3_).

3-((1*R*,5*S*,9*R*)-2-Phenethyl-9-propyl-2-azabicyclo[3.3.1]nonan-5-yl)phenol (**36**). The HCl salt of alkene **30** (250 mg,1 equiv, 628 µmol) was hydrogenated according to the general procedure using palladium on carbon (Escat 103) (6.68 mg, 0.1 equiv, 62.8 µmol) to afford **37** as a white foam (123 mg, 54% yield). The HCl salt of **37** was formed in *i*PrOH with 37% HCl (0.1 mL) and recrystallized from hot ethanol to give a white solid: mp 238–242 °C; ^1^H NMR (400 MHz; CD_3_OD): δ 7.36–7.30 (m, 4H), 7.29–7.23 (m, 1H), 7.15 (td, *J* = 7.9, 4.0 Hz, 1H), 6.86–6.79 (m, 2H), 6.65–6.63 (m, 1H), 3.78–3.71 (m, 1H), 3.66–3.44 (m, 4H), 3.18–3.08 (m, 2H), 2.49–2.44 (m, 1H), 2.40–2.29 (m, 1H), 2.20–1.85 (m, 7H), 1.42–1.22 (m, 3H), 1.22–0.98 (m, 2H), 0.89–0.74 (t, *J* = 7.2 Hz, 3H). ^13^C NMR (100 MHz; CD_3_OD): δ 158.7, 149.9, 137.8, 130.5, 129.99, 129.91, 128.3, 117.7, 114.3, 113.8, 59.4, 57.2, 51.1, 44.1, 39.5, 38.5, 31.8, 29.8, 28.8, 21.2, 20.9, 17.3, and 14.3; HRMS-ESI (*m*/*z*): [M + H^+^] calcd for: C_25_H_34_NO 364.2640; found 364.2643; Anal. Calcd. For C_25_H_34_ClNO·0.35 H_2_O: C, 73.9%; H, 8.61%; N, 3.45%. Found C, 73.56%; H, 8.25%; N, 3.18%; ${\left[\alpha \right]}_{20}^{D}$ 25.4° (c 0.7, CHCl_3_).

3-((1*R*,5*S*,9*R*)-9-Butyl-2-phenethyl-2-azabicyclo[3.3.1]nonan-5-yl)phenol (**37**). The HCl salt of alkene **31** (150 mg,1 equiv, 364 µmol) was hydrogenated according to the general procedure using palladium on carbon (Escat 103) (3.87 mg, 0.1 equiv, 36.4 µmol) to afford **37** as a white foam (125 mg, 90% yield). The tartrate salt of **37** was formed in *i*PrOH with L-tartaric acid and recrystallized from hot ethanol to give a white solid: mp 194–197 °C. ^1^H NMR (400 MHz; CD_3_OD): δ 7.36–7.31 (m, 4H), 7.26 (ddd, *J* = 8.9, 5.7, 2.9 Hz, 1H), 7.15 (t, *J* = 8.0 Hz, 1H), 6.87 (d, *J* = 7.2 Hz, 2H), 6.66–6.63 (m, 1H), 4.47 (s, 2H), 3.74 (s, 1H), 3.66–3.55 (m, 2H), 3.48 (t, *J* = 8.3 Hz, 2H), 3.18–3.10 (m, 2H), 2.53–2.38 (m, 2H), 2.17–2.02 (m, 4H), 1.99–1.86 (m, 3H), 1.34–1.05 (m, 6H), 0.80 (t, *J* = 6.9 Hz, 3H). ^13^C NMR (101 MHz; CD_3_OD): δ 177.1, 158.7, 150.2, 138.2, 130.5, 130.0, 129.9, 128.1, 117.8, 114.3, 113.9, 74.3, 59.3, 57.3, 50.6, 43.7, 39.2, 38.5, 31.8, 30.2, 28.9, 27.2, 23.6, 20.8, 17.7, 14.2. HRMS-ESI (*m*/*z*): [M + H^+^] calcd for: C_26_H_36_NO 378.2797; found 378.2800; C_30_H_41_NO_7_·0.1 H_2_O C: 68.06; H: 7.84; N: 2.65; found C: 67.73; H: 7.51; N: 2.59, ${\left[\alpha \right]}_{20}^{D}$ 38.0° (c 0.58, CHCl_3_).

*3*-((1*R*,5*S*,9*S*)-9-Ethyl-2-phenethyl-2-azabicyclo[3.3.1]nonan-5-yl)phenol (**38**). The HCl salt of alkene **32** (180 mg,1 equiv, 469 µmol) was dissolved in MeOH (15 mL) and hydrogenated according to the general procedure using palladium on carbon (Escat 103) (4.99 mg, 0.1 equiv, 46.9 µmol) to afford **38** and isolated as a white foam (160 mg, 88% yield). The HCl salt of **38** was formed in *i*PrOH with 37% HCl (0.1 mL) and recrystallized from ethanol to give a white solid: mp 249–253 °C; ^1^H NMR (400 MHz; CD_3_OD): δ 7.39–7.35 (m, 4H), 7.32–7.26 (m, 1H), 7.17 (t, *J* = 8.0 Hz, 1H), 6.81 (d, *J* = 7.9 Hz, 1H), 6.76 (t, *J* = 2.0 Hz, 1H), 6.65 (dd, *J* = 8.0, 2.2 Hz, 1H), 3.93 (s, 1H), 3.73–3.52 (m, 3H), 3.46–3.35 (m, 1H), 3.21 (td, *J* = 12.2, 5.5 Hz, 1H), 3.01 (td, *J* = 12.2, 4.9 Hz, 1H), 2.49–2.39 (m, 2H), 2.30–2.22 (m, 2H), 2.11–2.00 (m, 3H), 1.89–1.83 (m, 2H), 1.45–1.38 (m, 1H), 1.26 (ddd, *J* = 14.8, 7.5, 3.0 Hz, 1H), 0.91 (t, *J* = 7.4 Hz, 3H); ^13^C NMR (100 MHz; CD_3_OD): δ 158.8, 150.2, 137.5, 130.6, 130.0, 129.9, 128.4, 117.3, 114.2, 113.3, 56.5, 55.6, 51.7, 46.7, 42.0, 39.0, 31.5, 28.9, 25.1, 22.1, 19.2, 12.0; HRMS-ESI (*m*/*z*): [M + H^+^] calcd for: C_24_H_32_NO 350.2484; found: 350.2487; Anal. Calcd. For C_24_H_32_ClNO·0.1 H_2_O: C, 74.34%; H, 8.37%; N, 3.61%. Found C, 74.29%; H, 8.33%; N, 3.6%; ${\left[\alpha \right]}_{20}^{D}$ −18.3° (c 0.6, CHCl_3_).

3-((1*R*,5*S*,9*S*)-2-Phenethyl-9-propyl-2-azabicyclo[3.3.1]nonan-5-yl)phenol (**39**). The HCl salt of alkene **33** (325 mg,1 equiv, 817 µmol) was hydrogenated according to the general procedure using palladium on carbon (Escat 103) (8.69 mg, 0.1 equiv, 81.7 µmo to afford **39** as a white foam (220 mg, 74% yield). The HCl salt of **39** was formed in *i*PrOH with 37% HCl (0.1 mL) and recrystallized from hot ethanol to give a white solid: mp 253–256 °C. ^1^H-NMR (400 MHz; CD_3_OD): δ 7.37–7.33 (m, 4H), 7.29 (dq, *J* = 8.5, 4.2 Hz, 1H), 7.17 (dd, *J* = 10.4, 5.5 Hz, 1H), 6.82–6.80 (m, 1H), 6.76 (s, 1H), 6.66 (dd, *J* = 8.0, 1.8 Hz, 1H), 3.88 (s, 1H), 3.72–3.51 (m, 3H), 3.41 (td, *J* = 12.0, 5.0 Hz, 1H), 3.24–3.16 (m, 1H), 3.06–2.99 (m, 1H), 2.48–2.36 (m, 3H), 2.23 (dd, *J* = 14.1, 5.0 Hz, 1H), 2.12–1.99 (m, 3H), 1.89–1.81 (m, 2H), 1.51–1.38 (m, 2H), 1.24–1.05 (m, 2H), 0.86 (t, *J* = 7.0 Hz, 3H). ^13^C NMR (10 MHz; CD_3_OD): δ 158.8, 150.2, 137.5, 130.6, 130.02, 129.93, 128.4, 117.3, 114.2, 113.4, 56.4, 56.2, 51.7, 44.6, 41.9, 38.9, 31.5, 28.9, 28.5, 25.1, 22.1, 21.5, and 14.2. HRMS-ESI (*m*/*z*): [M + H^+^] calcd for: C_25_H_34_NO 364.2640; found 364.2637; Anal. Calcd. For C_25_H_34_ClNO·0.05 H_2_O; C: 74.9%; H: 8.57%; N: 3.49%; found: C: 74.97%, H: 8.73%; N: 3.49%; ${\left[\alpha \right]}_{20}^{D}$ −27.7° (c 0.35, CHCl_3_).

3-((1*R*,5*S*,9*S*)-9-Butyl-2-phenethyl-2-azabicyclo[3.3.1]nonan-5-yl)phenol (**40**). The HCl salt of alkene **34** (150 mg,1 equiv, 364 µmol) was hydrogenated according to the general procedure using palladium on carbon (Escat 103) (3.87 mg, 0.1 equiv, 36.4 µmol) hydrogenated according to the general procedure using to afford **40** as a white foam (120 mg, 87% yield). The HCl salt of **40** was formed in *i*PrOH (0.5 mL) with 37% HCl (0.05 mL) and recrystallized from ethanol to give a white solid: mp 254–259 °C. ^1^H NMR (400 MHz; CD_3_OD): δ 7.37–7.26 (m, 5H), 7.15 (dt, *J* = 10.4, 6.5 Hz, 1H), 6.81 (d, *J* = 8.0 Hz, 1H), 6.76 (s, 1H), 6.71–6.62 (m, 1H), 3.88 (s, 1H), 3.70–3.49 (m, 4H), 3.46–3.37 (m, 1H), 3.20 (td, *J* = 12.1, 5.3 Hz, 1H), 3.02 (td, *J* = 12.1, 5.1 Hz, 1H), 2.48–2.33 (m, 3H), 2.22 (dt, *J* = 14.0, 6.6 Hz, 1H), 2.05 (t, *J* = 8.5 Hz, 3H), 1.87 (q, *J* = 11.2 Hz, 2H), 1.49–1.33 (m, 2H), 1.29–1.10 (m, 4H), 0.85 (t, *J* = 7.2 Hz, 3H). 13-C NMR (101 MHz; CD_3_OD): δ 158.7, 150.2, 130.6, 130.01, 129.93, 128.4, 117.4, 114.2, 113.4, 56.44, 56.28, 51.7, 44.9, 41.9, 38.9, 31.5, 30.7, 28.9, 26.1, 25.2, 23.5, 22.1, and 14.3. HRMS-ESI (*m*/*z*): [M + H^+^] calcd for: C_26_H_36_NO 378.2797; found 378.2800; C_26_H_36_ClNO ·0.35 C_2_H_6_O calc. C: 74.55%; H: 8.93%; N: 3.26%; found C: 74.65%, H: 9.06%; N: 3.37%; ${\left[\alpha \right]}_{20}^{D}$ −17.9° (c 0.75, CHCl_3_).

tertert-Butyl (1*S*,5*S*)-5-(3-methoxyphenyl)-9-oxo-2-azabicyclo[3.3.1]nonane-2-carboxylate (**44**). To a vial containing **1** (2.5 g, 1 equiv, 9.6 mmol) in acetonitrile (15 mL), under argon, was added potassium carbonate (2.7 g, 2.0 equiv, 19 mmol) and cyanogen bromide (3.9 mL, 2.0 equiv, 19 mmol). The reaction mixture was stirred for 2 h at room temperature followed by 2 h at reflux. Upon completion, the reaction mixture was filtered through Celite, washed several times with CHCl_3_, and the filtrate was concentrated in vacuo. The resulting oil was taken up in MeOH (3.0 mL) and treated with 3 M HCl (29 mL). The reaction mixture was stirred at reflux for 16 h and subsequently quenched with 7 N NH_4_OH in MeOH, extracted with CHCl_3_ and concentrated in vacuo. To the crude solution was added dry dichloromethane (12 mL) at 0 °C, di-tert-butyl dicarbonate (2.9 g, 1.4 equiv, 13 mmol), triethylamine (1.9 mL, 1.4 equiv, 13 mmol), and 4-dimethylaminopyridine (0.12 g, 0.1 equiv, 0.96 mmol) dropwise. The solution was allowed to stir under argon for 30 min at 0 °C then warmed to room temperature. After 1 h, TLC showed consumption of starting material. Saturated ammonium chloride was added, and the mixture was extracted with CH_2_Cl_2_, washed with brine, and dried over sodium sulfate. The crude mixture was loaded onto silica and purified via flash chromatography eluting with 0–30% ethyl acetate in hexane.

tert-Butyl (1*S*,5*S*)-5-(3-methoxyphenyl)-9-methylene-2-azabicyclo[3.3.1]nonane-2-carboxylate (**45**). To a vial containing ketone **44** (1.85 g, 1 equiv, 5.36 mmol) in THF (25.0 mL) at 0 °C was added Tebbe’s Reagent (10.7 mL, 1 equiv, 5.36 mmol). The resulting mixture was stirred at 0 °C for 1 h and then slowly warmed up to room temperature for an additional 4 h. Upon completion by TLC, the reaction was cooled to 0 °C and 50 mL of Et_2_O was added. The reaction was quenched carefully with 1.8 N NaOH. A very vigorous gas evolution took place and a thick red/orange precipitate formed. Magnesium sulfate was added, and the mixture was allowed to stir an additional 5 min. The solids were filtered and washed with EtOAc. Flash column chromatography using 1–5% CMA in CHCl_3_ yielded **45** as an orange foam (0.81 g, 44% yield).

tert-Butyl (1*S*,5*R*,9*R*)-5-(3-methoxyphenyl)-9-methyl-2-azabicyclo[3.3.1]nonane-2-carboxylate (**46**). Methylene **45** (810 mg,1 equiv, 2.13 mmol) was hydrogenated according to the general procedure using palladium on carbon (Escat 103) (22.7 mg, 0.1 equiv, 213 µmol) to afford **46** as a white foam (700 mg, 95% yield). The reaction gave a mixture of epimers that were used without further purification.

(1*S*,5*R*,9*R*)-5-(3-Methoxyphenyl)-9-methyl-2-phenethyl-2-azabicyclo[3.3.1]nonane (**47**). To a solution of **46** (1 g, 1 equiv, 3 mmol) in dichloromethane (30 mL) at 0 °C was added trifluoroacetic acid (2 mL, 10 equiv, 0.03 mol), dropwise. After 15 min, the reaction was allowed to warm to room temperature and allowed to stir for 1 h. Upon completion as determined using TLC, the reaction mixture was cooled to 0 °C and quenched with NaHCO_3_ and extracted with dichloromethane. The crude oil was taken up in acetonitrile (32 mL), was treated with K_2_CO_3_, and the mixture was purged with argon. (2-Bromoethyl)benzene (0.8 g, 1.5 equiv, 4 mmol) was added, and the reaction was refluxed under argon for 16 h. Upon completion, the reaction mixture was concentrated in vacuo then extracted with CHCl_3_. The organic extracts were washed with water and brine, dried with sodium sulfate and concentrated in vacuo. Purification by flash column chromatography on silica using 0–100% EtOAc: Hexanes to isolate **47** as a yellow oil (830 mg, 80% yield). The resulting oil was a mixture of epimers and was used without further purification.

3-((1*S*,5*R*,9*R*)-9-Methyl-2-phenethyl-2-azabicyclo[3.3.1]nonan-5-yl)phenol (**48**). In an oven-dried round-bottom flask, **47** (600 mg, 1 equiv, 2.9 mmol) was suspended in dichloromethane (30 mL) and the mixture was cooled to −78 °C. Tribromoborane (0.3 mL, 2 equiv, 5.8 mmol) was added dropwise and the reaction was stirred at −78 °C. The reaction mixture was allowed to warm to room temperature and stirred 2 h. Upon completion, the reaction mixture was cooled to 0 °C and quenched with 15 mL MeOH dropwise and stirred for 30 min. A total of 20 mL 1N HCl was added, and the reaction mixture was refluxed at 100 °C for 1 h. The reaction mixture was then cooled to 0 °C and made basic (pH > 10.5) with NH_4_OH and extracted with 9:1 CHCl_3_:MeOH. The combined organic layers were washed with water and brine, dried with sodium sulfate, and concentrated in vacuo. Purification by silica gel flash chromatography using 10–100% EtOAc Hexanes resulted in a tan foam (420 mg, 73%). The HCl salt of **48** was formed in *i*PrOH with 37% HCl (0.1 mL) and recrystallized from ethanol to give a white solid: mp 264–269 °C. ^1^H NMR (400 MHz; CD_3_OD): δ 7.39–7.34 (m, 4H), 7.29 (m, 1H), 7.17 (t, *J* = 7.9 Hz, 1H), 6.81 (d, *J* = 7.9 Hz, 1H), 6.77 (t, *J* = 1.9 Hz, 1H), 6.65 (dd, *J* = 8.0, 2.3 Hz, 1H), 3.77 (s, 1H), 3.71–3.59 (m, 2H), 3.52 (td, *J* = 12.2, 5.5 Hz, 1H), 3.40 (td, *J* = 12.1, 5.2 Hz, 1H), 3.16 (td, *J* = 12.2, 5.4 Hz, 1H), 3.03 (td, *J* = 12.2, 5.2 Hz, 1H), 2.66–2.60 (m, 1H), 2.54–2.38 (m, 2H), 2.24 (dd, *J* = 14.5, 5.0 Hz, 1H), 2.13–1.98 (m, 3H), 1.91–1.82 (m, 2H), 0.97 (d, *J* = 7.3 Hz, 3H); ^13^C NMR (100 MHz; CD_3_OD): δ 158.8, 150.1, 137.6, 130.6, 130.02, 129.91, 128.4, 117.3, 114.2, 113.3, 60.6, 56.6, 51.6, 41.9, 39.3, 38.4, 31.5, 28.1, 25.0, 22.2, and 13.7; HRMS-ESI (*m*/*z*): [M + H]^+^ calcd. for C_23_H_29_NO 336.2327, found 336.2321; Calcd for C_23_H_30_ClNO·0.05 H_2_O: C, 74.09%; H, 8.14%; N, 3.76%. Found C: 74.18% H: 8.07% N: 3.72%; ${\left[\alpha \right]}_{20}^{D}$ 33.3° (c 1.4, CHCl_3_).

3-((1*R*,5*S*,9*S*)-9-Methyl-2-phenethyl-2-azabicyclo[3.3.1]nonan-5-yl)phenol (**56**). In an oven-dried round-bottom flask, **55** (400 mg, 1 equiv, 1.14 mmol) was suspended in dichloromethane (20 mL) and the mixture was cooled to −78 °C. Tribromoborane (0.2 mL, 2 equiv, 2.29 mmol) was added dropwise and the reaction was stirred at −78 °C. The reaction mixture was allowed to warm to room temperature and stirred 2 h. Upon completion, the reaction mixture was cooled to 0 °C and quenched with 15 mL MeOH dropwise and stirred for 30 min. A total of 20 mL 1N HCl was added, and the reaction mixture was refluxed at 100 °C for 1 h. The reaction mixture was then cooled to 0 °C and made basic (>10.5) with NH_4_OH and extracted with 9:1 CHCl_3_:MeOH. The combined organic layers were washed with water and brine, dried with sodium sulfate, and concentrated. Purification by silica gel flash chromatography 10–100% EtOAc Hexanes resulted in a tan foam (380 mg, 99%). The HCl salt was isolated as a white solid (380 mg, 99% yield): mp 268–272 °C. ^1^H NMR (400 MHz; CD_3_OD): δ 7.39–7.33 (m, 4H), 7.29 (ddd, *J* = 9.1, 5.9, 2.7 Hz, 1H), 7.17 (t, *J* = 8.0 Hz, 1H), 6.81 (d, *J* = 7.9 Hz, 1H), 6.76 (t, *J* = 2.0 Hz, 1H), 6.65 (dd, *J* = 8.0, 2.0 Hz, 1H), 3.77 (d, *J* = 0.6 Hz, 1H), 3.72–3.58 (m, 2H), 3.56–3.49 (m, 1H), 3.44–3.37 (m, 1H), 3.19–3.12 (m, 1H), 3.02 (ddd, *J* = 14.6, 9.9, 4.9 Hz, 1H), 2.66–2.61 (m, 1H), 2.54–2.39 (m, 2H), 2.27–2.22 (m, 1H), 2.13–1.98 (m, 3H), 1.91–1.82 (m, 2H), 0.96 (d, *J* = 7.3 Hz, 3H). ^13^C NMR (101 MHz; CD_3_OD): δ 158.8, 150.1, 137.6, 130.6, 130.03, 129.89, 128.4, 117.3, 114.2, 113.3, 60.7, 56.6, 51.6, 41.9, 39.3, 38.4, 31.5, 28.1, 25.0, 22.2, and 13.6; HRMS-ESI (*m*/*z*): [M + H]^+^ calcd. for C_23_H_29_NO 336.2327, found 336.2326; Calcd for C_23_H_30_ClNO: C, 74.27%; H, 8.13%; N, 3.77%. Found C: 74.39% H: 8.27% N: 3.82%; %; ${\left[\alpha \right]}_{20}^{D}$ −34.8° (c 0.79, CHCl_3_).

### 3.3. In Vitro Assay

#### 3.3.1. Cell Lines and Cell Culture

Cell lines and cell culture: cAMP Hunter^TM^ Chinese hamster ovary cells (CHO-K1) that express human μ-opioid receptor (OPRM1), human κ-opioid receptor (OPRMK1), and human δ-receptor (OPRMD1) were purchased from Eurofins DiscoverX (Fremont, CA, USA) and used for the forskolin-induced cAMP accumulation assays [16]. All cell lines were maintained in F-12 media with 10% fetal bovine serum (Life Technologies, Grand Island, NY, USA), 1% penicillin/streptomycin/l-glutamine (Life Technologies), and 800 µg/mL Geneticin (Mirus Bio, Madison, WI, USA). All cells were grown at 37 °C and 5% CO_2_ in a humidified incubator.

#### 3.3.2. Forskolin-Induced cAMP Accumulation Assays

Briefly, cells were plated at 10,000 cells/well density in a 384-well tissue culture plate and incubated overnight at 37 °C in 5% CO_2_. Stock solutions of compound were made in 100% DMSO at a 5 mM concentration. A serial dilution of 10 concentrations was made using 100% DMSO, creating 100X working solutions of the compound for treatment. The 100X working solutions were then diluted to 5X working solutions using assay buffer consisting of Hank’s Buffered Salt Solution, HEPES, and forskolin. For the agonist assay, cells were incubated at 37 °C with compounds for 30 min at a 1X final concentration. For the antagonist assay, cells were incubated at 37 °C with compounds for 15 min before 30-min incubation at 37 °C with selected agonist at their EC_50_ or EC_90_ dose. Detection was made by using the HitHunter cAMP Assay for Small Molecules by DiscoverX according to manufacturer’s directions, and the BioTek Synergy H1 hybrid plate reader (BioTek, Winooski, VT, USA) and Gen5 Software version 2.01 were used to quantify luminescence (BioTek, Winooski, VT, USA) [16].

## 4. Conclusions

Four series of C9-alkyl 5-phenylmorphans have been synthesized. Eight of these diastereomers were more potent than morphine (C9-methyl compounds **51** and **56**, C9-ethyl compounds **15**, **18**, **35**, and **38**, and the C9-propyl compounds **16**, and **36**). Among them, compounds **51**, **18**, **35**, and **36** showed full or close to full MOR agonism. One C9-methyl compound, **48**, was a potent antagonist, more potent, selective, and with higher antagonist efficacy than naltrexone. The MOR antagonist **48** did not interact at all with DOR or KOR as an agonist and it had very little DOR antagonist activity. It was about as potent as the reference kappa ligand, nor-BNI, as a KOR antagonist. It will be further evaluated in the future in vivo as a possible replacement for naloxone in treating respiratory depression caused by the more potent agonists that are currently being abused.

## 5. Patents 

(1) K. C. Rice. A. E. Jacobson, A. Sulima, and D. C. Chambers, “Selective Opioid Receptor Agonists and Antagonists”, US Patent Application Serial No. 63/393,035, filed 28 July 2022; (2) Rice, K. C.; Jacobson, A. E.; LI, F.; Gutman, E. S.; Bow, E. W. Biased potent opioid-like agonists as improved medications to treat chronic and acute pain andmethods of using the same. US Application Serial No.: 62/644,791 filed on 19 March 2018. US Patent 11,352,365 issued 6-7-2022. International Publication WO 2019/182950 A1 26 September 2019.

## Figures and Tables

**Table 1:** Selectivity of hydrogenation reactions based on R_1_ and R_2_ groups.

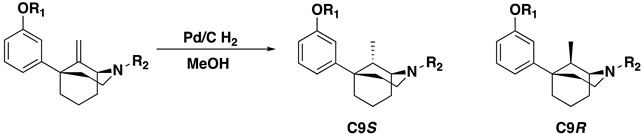		
Reaction	Starting Material	C9*S*:C9*R* Ratio
1	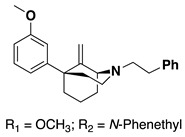	1:0
2	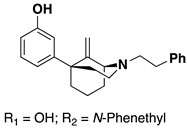	5:1
3	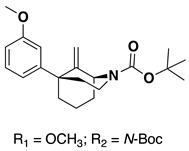	1:3

**Table 2:** Opioid Receptor Activity Measured in the Forskolin-induced cAMP Accumulation Assay ^a^.

Compd#	Molecular Structure	MOR		DOR		KOR	
		Agonist	Antagonist ^b^	Agonist	Antagonist ^c^	Agonist	Antagonist ^d^
		EC_50_ ± SEM (nM) (%E_max_ ± SEM)	IC_50_ ± SEM (nM) (%I_max_ ± SEM)	EC_50_ ± SEM (nM) (%E_max_ ± SEM)	IC_50_ ± SEM (nM) (%I_max_ ± SEM)	EC_50_ ± SEM (nM) (%E_max_ ± SEM)	IC_50_ ± SEM (nM) (%E_max_ ± SEM)
**43** ^e^ (−)-ACH-1-136	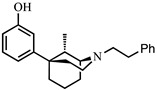	16 ± 3 (78 ± 5%)	N/D	699 ± 388 (51 ± 8%)	N/D	4.6 ± 2.5 (21 ± 6%)	369 ± 187 (74 ± 3%)
**51** ^e^ (+)-ACH-1-135	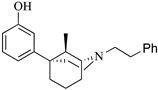	1.9 ± 0.5 (96 ± 2%)	N/D	>10,000	151 ± 67 (97 ± 9%)	>10,000	54 ± 14 (94 ± 2%)
**48** DC-01-0117 1*S*,5*R*,9*R*	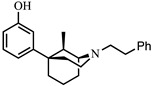	>10,000	3.9 ± 0.9 (153 ± 12%)	>10,000	194 ± 48 (144 ± 5%)	>10,000	2.6 ± 0.5 (106 ± 8%)
**56** DC-01-0123 1*R*,5*S*,9*S*	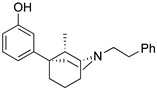	0.8 ± 0.4 (39 ± 8%)	6.6 ± 0.4 (93 ± 7%)	>10,000	489 ± 147 (196 ± 23%)	>10,000	14 ± 7 (92 ± 4%)
**15** DC-01-0105 1*S*,5*R*,9*R*	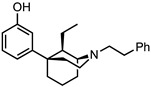	1.4 ± 0.7 (48 ± 1%)	3.3 ± 0.9 (69 ± 7%)	>10,000	75 ± 30 (157 ± 11%)	>10,000	11 ± 6 (112 ± 9%)
**18** DC-01-0106 1*S*,5*R*,9*S*	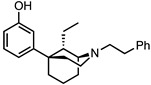	3.4 ± 1.5 (97 ± 1%)	N/D	426 ± 148 (69 ± 9%)	N/D	22 ± 8 (47 ± 2%)	N/D
**35** DC-01-0079 1*R*,5*S*,9*R*	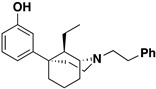	1.0± 0.5 (96 ± 2%)	N/D	2.9 ± 0.9 (29 ± 2%)	42 ± 9 (110 ± 5%)	>10,000	112 ± 57 (92 ± 7%)
**38** DC-01-0082 1*R*,5*S*,9*S*	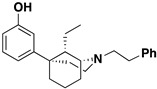	4.9 ± 1.2 (31 ± 3%)	35± 5 (92 ± 7%)	>10,000	435 ± 132 (149 ± 38%)	>10,000	48 ± 21 (91± 4%)
**16** EG-1-203 (Published and retested) 1*S*,5*R*,9*R*	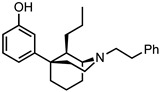	1.4 ± 0.4 (46 ± 6%)	11 ± 3 (48 ± 1%)	>10,000	84 ± 33 (96 ± 4%)	>10,000	12 ± 5 (94± 6%)
**19** DC-01-0096 1*S*,5*R*,9*S*	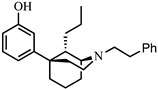	36 ± 19 (45 ± 4%)	415 ± 24 (101 ± 9%)	>10,000	990 ± 389 (112± 10%)	106 ± 31 (61 ± 4%)	N/D
**36** DC-01-0098 1*R*,5*S*,9*R*	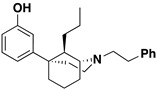	3.2 ± 0.9 (94 ± 2%)	N/D	>10,000	162 ± 47 (147± 28%)	>10,000	204 ± 77 (95 ± 5%)
**39** DC-01-0097 1*R*,5*S*,9*S*	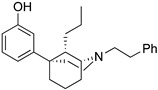	7.3 ± 2.2 (24 ± 1%)	140 ± 19 (123 ± 12%)	>10,000	368 ± 54 (170± 6%)	>10,000	54 ± 26 (102± 2%)
**17** DC-01-0107 1*S*,5*R*,9*R*	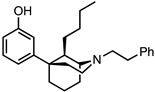	7.2 ± 3.5 (32 ± 5%)	3.8 ± 0.8 (80 ± 6%)	>10,000	30 ± 9 (125 ± 8%)	>10,000	5.2 ± 0.6 (103 ± 15%)
**20** DC-01-0108 1*S*,5*R*,9*S*	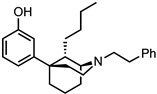	880 ± 245 (66 ± 3%)	1369 ± 823 (59 ± 4%)	>10,000	2078 ± 1056 (122 ± 21%)	638± 157 (87± 9%)	N/D
**37** DC-01-0118.2 1*R*,5*S*,9*R*	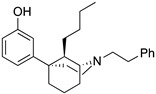	53 ± 8 (97± 2%)	N/D	>10,000	107 ± 27 (159 ± 13%)	>10,000	>10,000
**40** DC-01-0131 1*R*,5*S*,9*S*	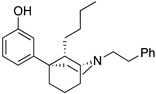	55 ± 4 (56 ± 4%)	2089 ± 1106 (101 ± 7%)	>10,000	681 ± 73 (178 ± 15%)	>10,000	604 ± 234 (102 ± 6%)
**Standards**							
	Morphine	5.8 ± 0.3 (102 ± 0.1%)		525 ± 94 (75 ± 5%)		346 + 39 (86 ± 5%)	
	Naltrexone	2.1 ± 1.2 (30 ± 6%)	11 ± 0.9 (104± 0.6%)	>10,000	295 ± 48 (99 ± 1%)	0.6± 0.3 (57± 7%)	5.5 ± 1.0 (41 ± 7%)
	U50488H					0.31 ± 0.03 (99.5 ± 0.3%)	
	SNC80			1.7 ± 0.2 (79 ± 2%)			
	nor-BNI						2.3 ± 0.3 (102 ± 1%)
	Naltrindole				1.1 ± 0.2 (107 ± 5%)		

^a^ Inhibition of forskolin-induced cAMP accumulation; cAMP Hunter^TM^ Chinese hamster ovary cells (CHO-K1) that express human μ-opioid receptor (OPRM1), human κ-opioid receptor (OPRK1), and human δ-opioid receptor (OPRD1) were used for the forskolin-induced cAMP accumulation assay in order to determine potency and efficacy of the compounds following the previously established methods; [17] to determine % efficacy in forskolin-induced cAMP assays, data were blank subtracted with the vehicle control, followed by normalization to the forskolin control. Data were then analyzed in GraphPad Prism 8 (GraphPad, LaJolla, CA, USA) using nonlinear regression; values are expressed as the mean ± SEM of at least three independent experiments; N/D = not determined. ^b^ MOR Antagonist potency (IC_50_) determined versus EC90 of fentanyl; Degree of antagonism (I_max_) normalized to naltrexone. ^c^ DOR Antagonist potency (IC_50_) determined versus EC50 of SNC80; degree of antagonism (I_max_) normalized to naltrexone. ^d^ KOR Antagonist potency (IC_50_) determined versus EC90 of U50488H; degree of antagonism (Imax) normalized to *nor*-BNI. ^e^ Structure previously published [11]. Data from re-assay.

## Data Availability

The data presented in this study are available in this article or in the supplementary material.

## Supplementary Materials

The following supporting information can be downloaded at https://www.mdpi.com/article/10.3390/molecules28145411/s1, Figures S1–S12: ^1^H and ^13^C-NMR spectra of novel compounds are shown.

## Abbreviations

MOR: μ-opioid receptor; DOR, δ-opioid receptor; KOR, κ-opioid receptor; cAMP, cyclic adenosine monophosphate.
