# Supplementary material for: A MOR Antagonist with High Potency and Antagonist Efficacy among Diastereomeric C9-Alkyl-Substituted N-Phenethyl-5-(3-hydroxy)phenylmorphans
Source: Molecules. 2023 Jul 14;28(14):5411. doi: 10.3390/molecules28145411 (PMC10386414; doi:10.3390/molecules28145411)
Supplement: Supplementary file 1 [file molecules-28-05411-s001.zip › molecules-2481220-supplementary.pdf]

Supplementary Materials

A MOR Antagonist with High Potency and Antagonist Efficacy Among Diastereomeric C9-Alkyl-Substituted *N*-Phenethyl-5-(3-hydroxy)phenylmorphans

Dana R. Chambers[1], Agnieszka Sulima[1], Dan Luo[2], Thomas E. Prisinzano[2], Arthur E. Jacobson,[1,*] Kenner C. Rice[1,*]

[1]Drug Design and Synthesis Section, Molecular Targets and Medications Discovery Branch, Intramural Research Program, National Institute on Drug Abuse and the National Institute on Alcohol Abuse and Alcoholism, National Institutes of Health, Department of Health and Human Services, 9800 Medical Center Drive, Bethesda, MD 20892-3373, United States

[2]Department of Pharmaceutical Sciences, College of Pharmacy, University of Kentucky, 789 S. Limestone Street, Lexington, Kentucky 40536, USA

*Correspondence: arthurj@nida.nih.gov (A.E.J.); kennerr@nida.nih.gov (K.C.R.)

Tel.: +1-301-451-5028 (A.E.J.); +1-301-451-4799 (K.C.R.)

Table of Contents

**Figure**		**Page #**
S1-S12	^{1}H (top of page) & ^{13}C NMR (bottom of page)	3 - 14

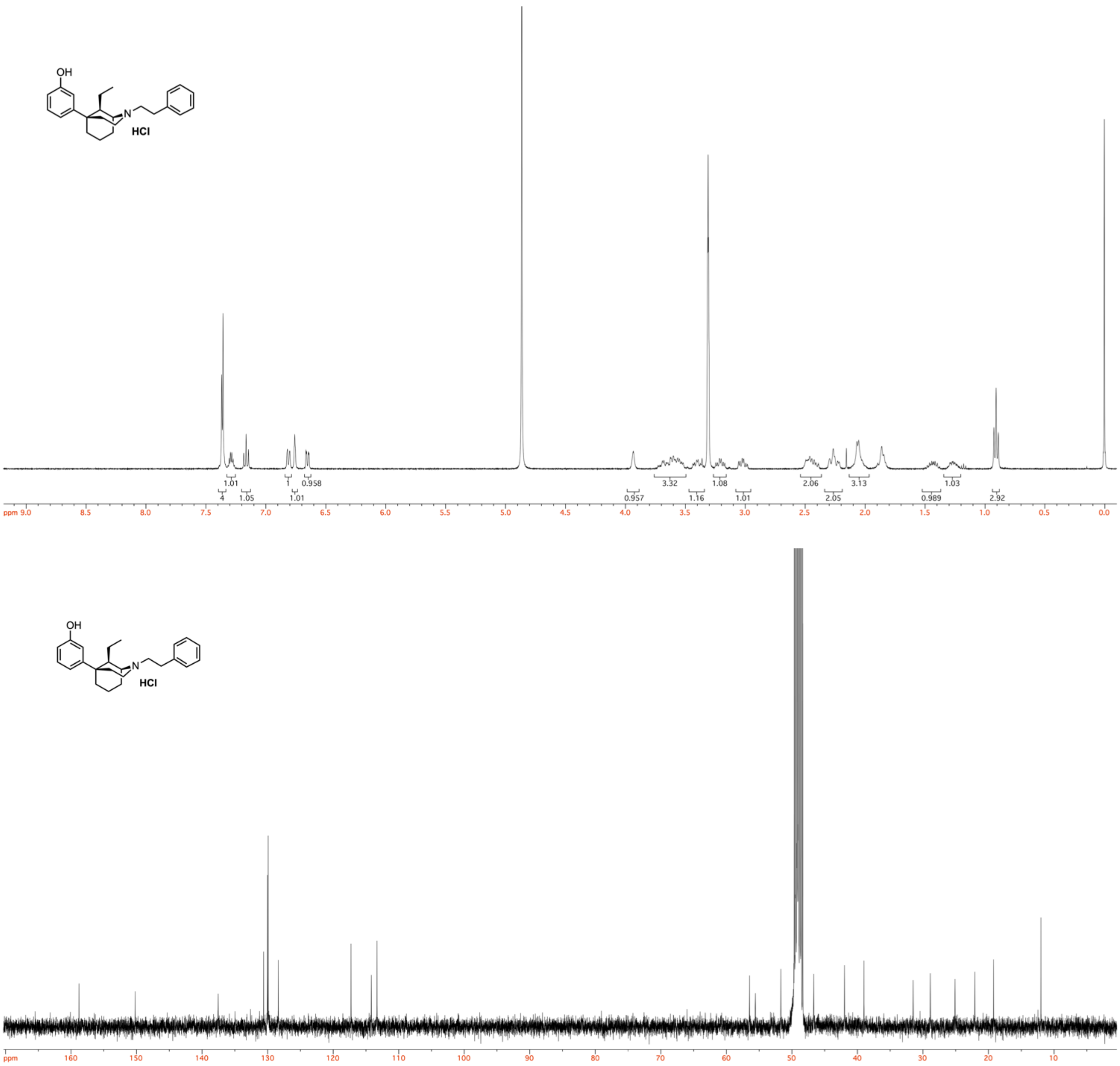

Figure S1: ^{1}H and ^{13}C-NMR spectra of compound **15** (C9*R*)

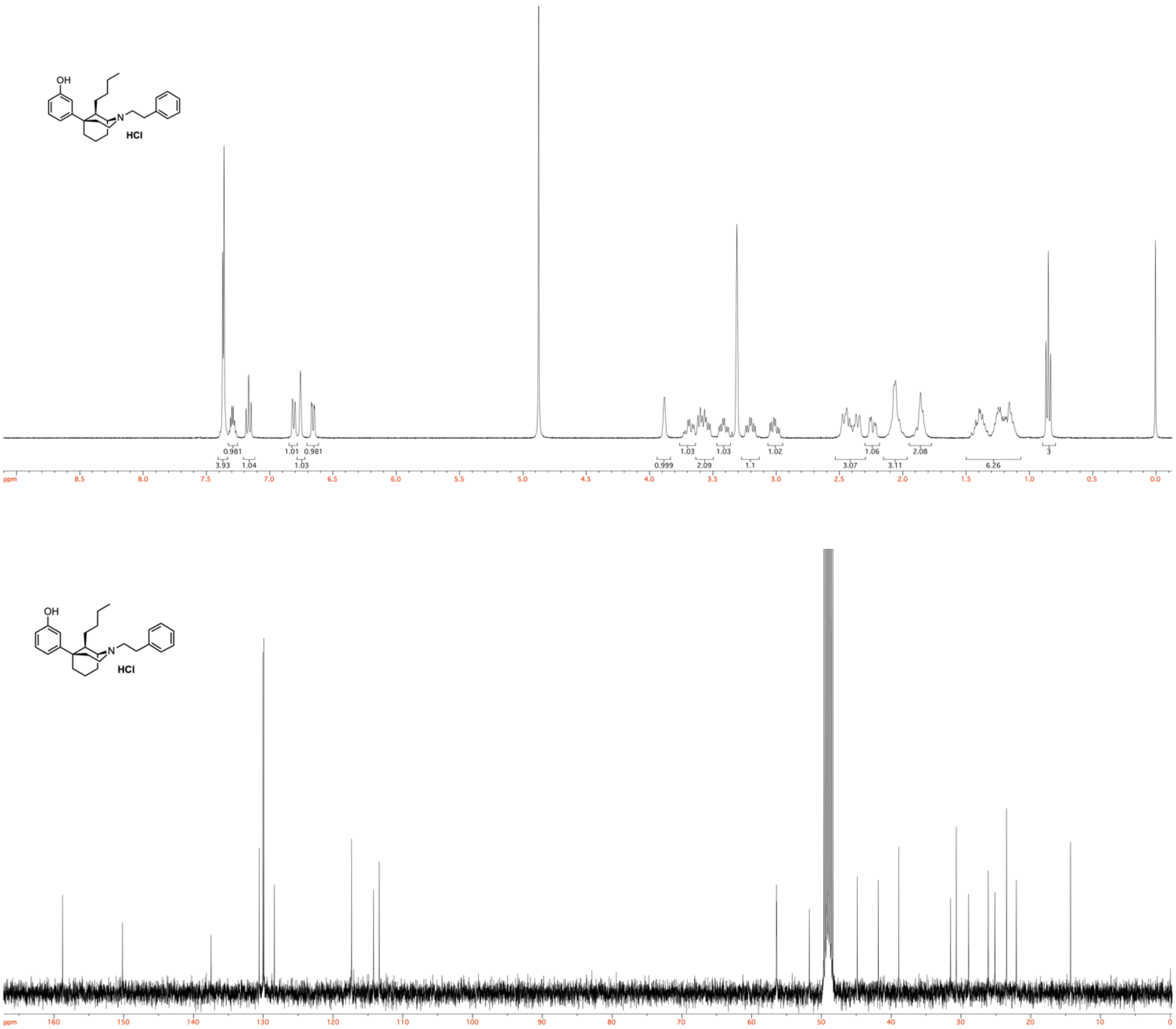

Figure S2: ^{1}H and ^{13}C-NMR spectra of compound **17** (C9*R*)

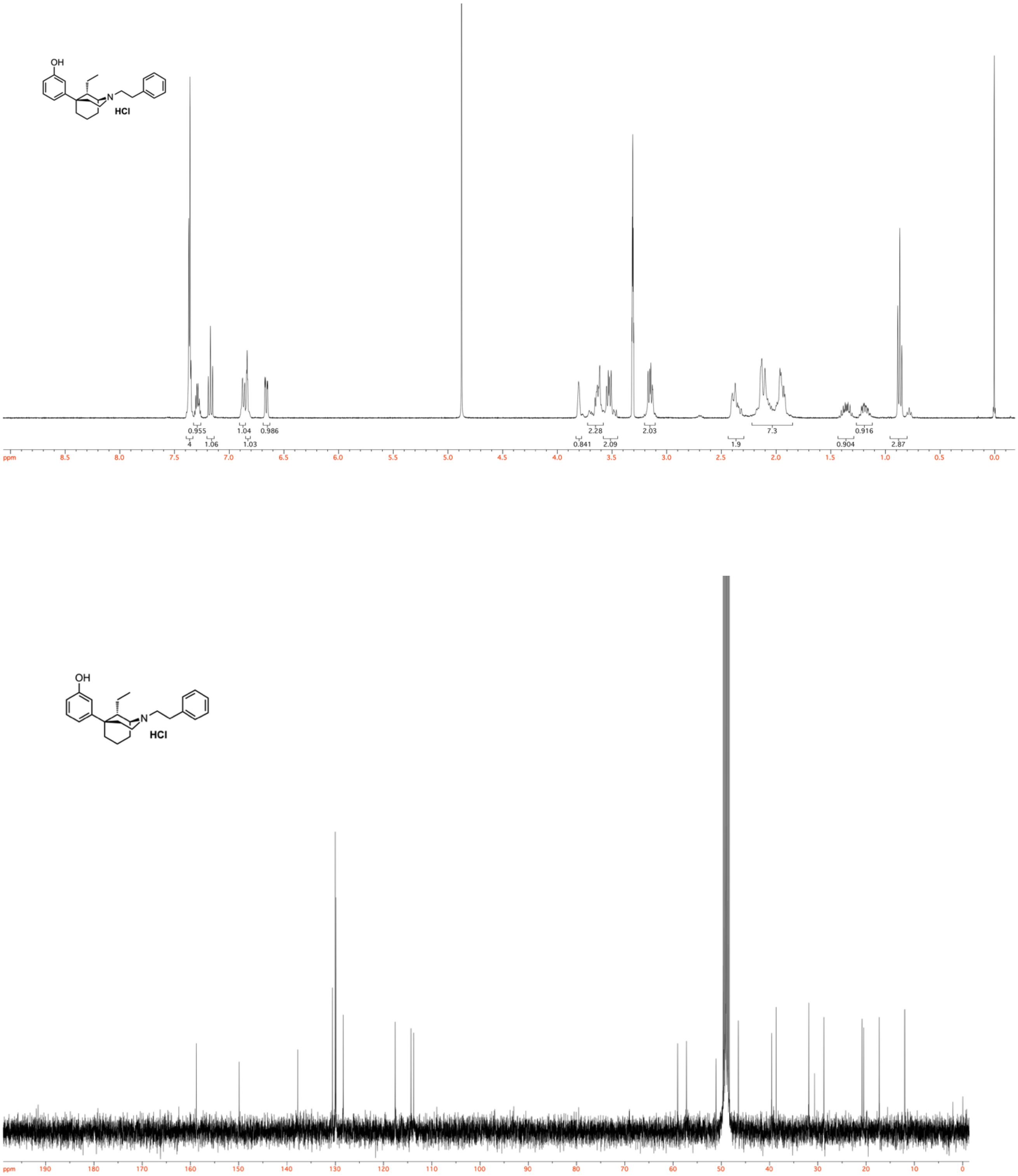

Figure S3: ^{1}H and ^{13}C-NMR spectra of compound **18** (C9*S*)

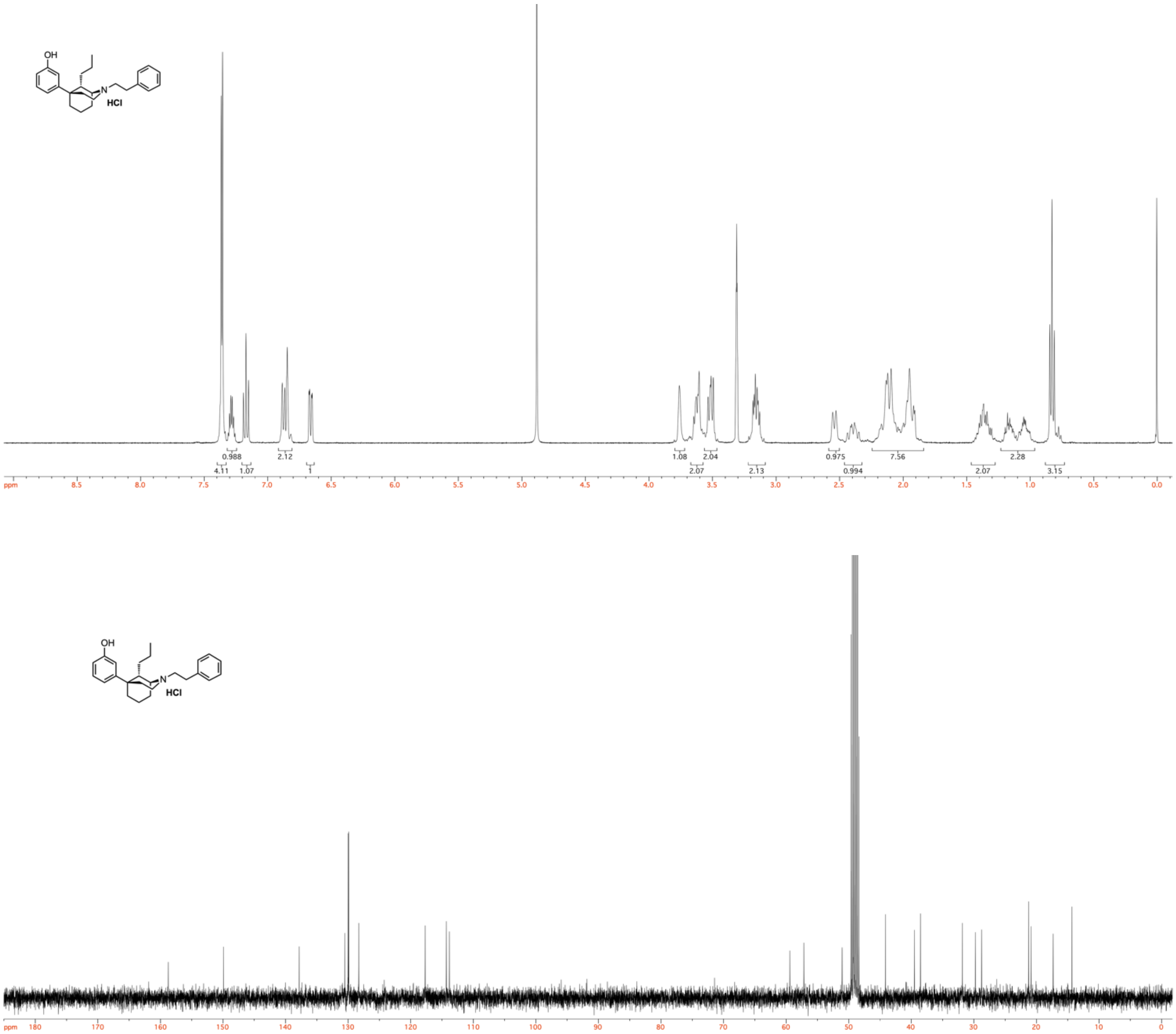

Figure S4: ^{1}H and ^{13}C-NMR spectra of compound **19** (C9*S*)

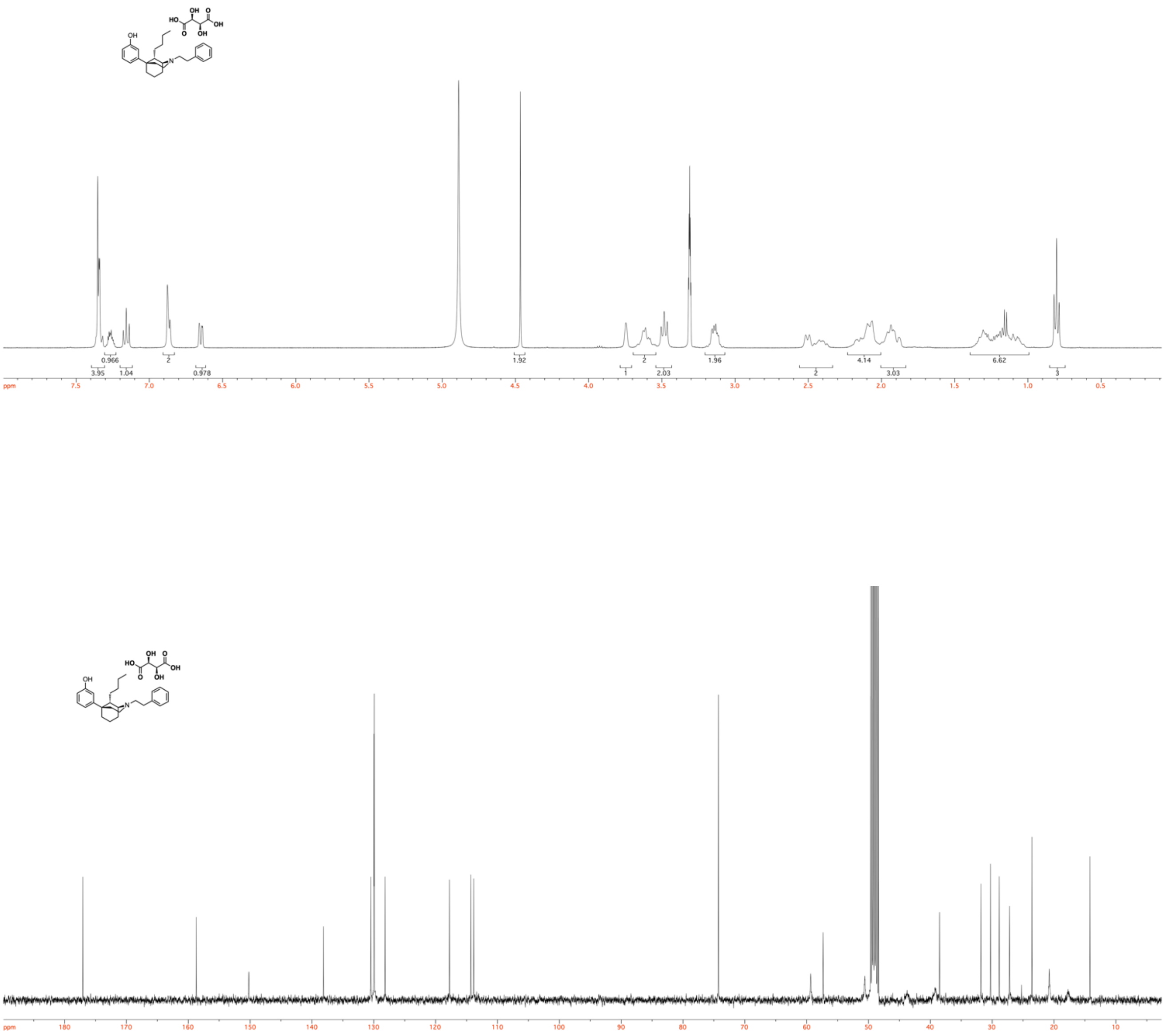

Figure S5: ^{1}H and ^{13}C-NMR spectra of compound **20** (C9*S*)

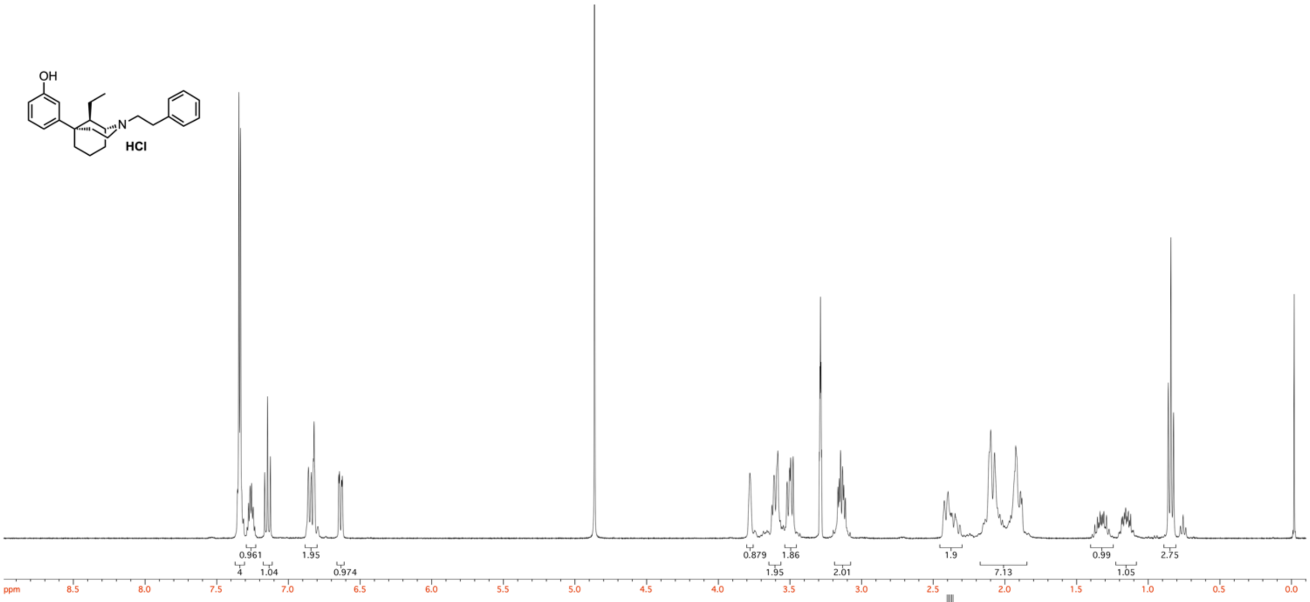

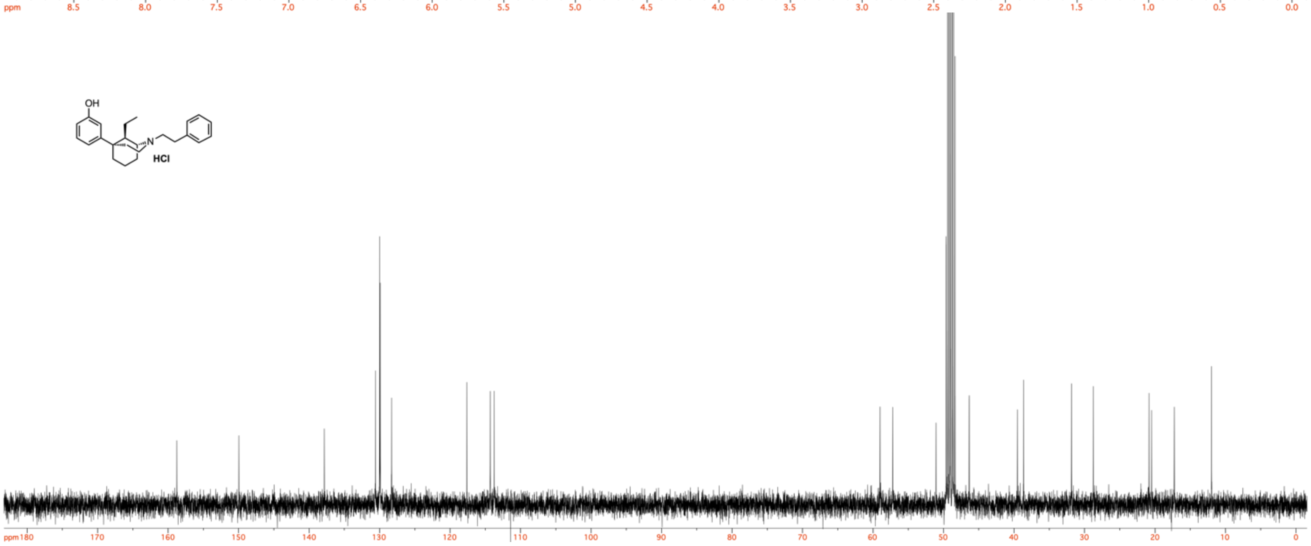

Figure S6: ^{1}H and ^{13}C-NMR spectra of compound **35** (C9*R*)

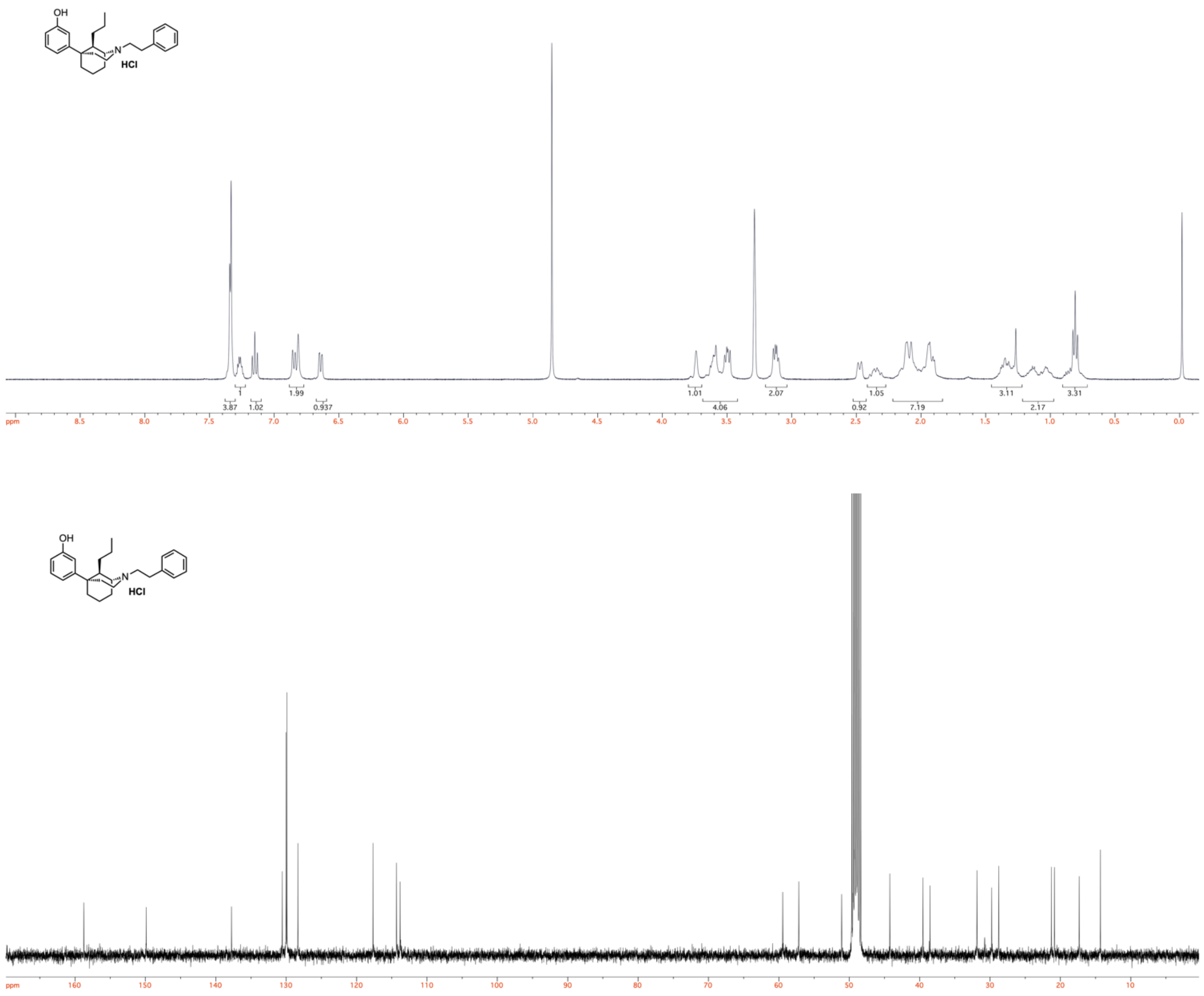

Figure S7: ^{1}H and ^{13}C-NMR spectra of compound **36** (C9*R*)

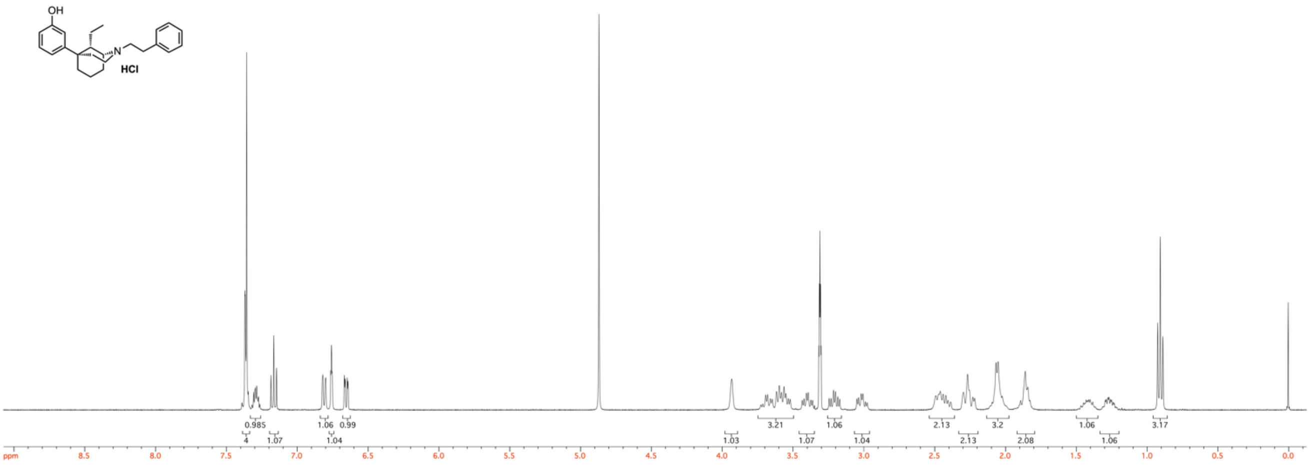

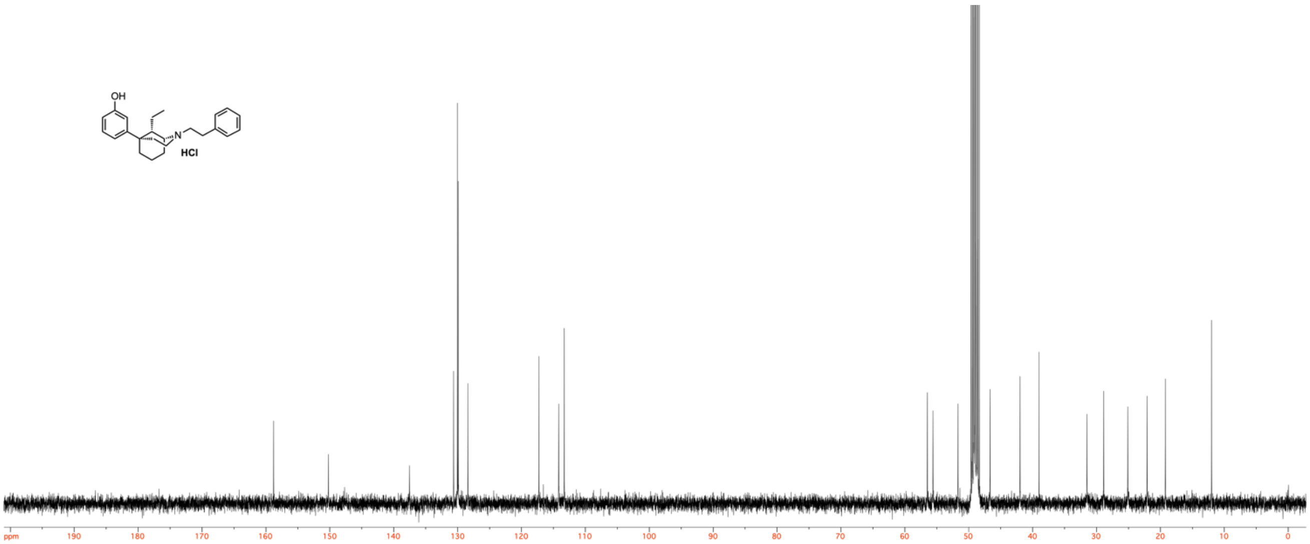

Figure S8: ^{1}H and ^{13}C-NMR spectra of compound **38** (C9*S*)

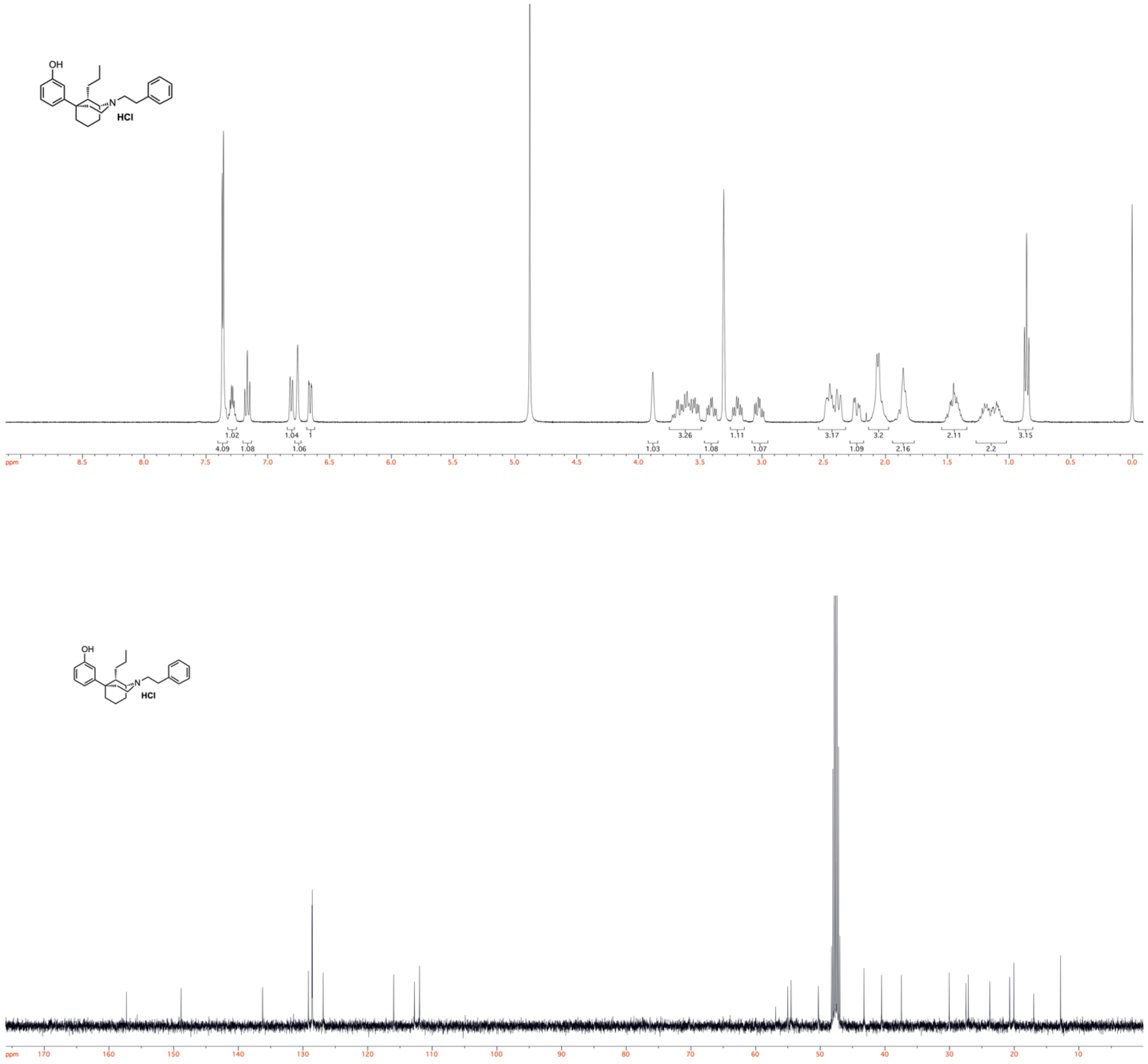

Figure S9: ^{1}H and ^{13}C-NMR spectra of compound **39** (C9*S*)

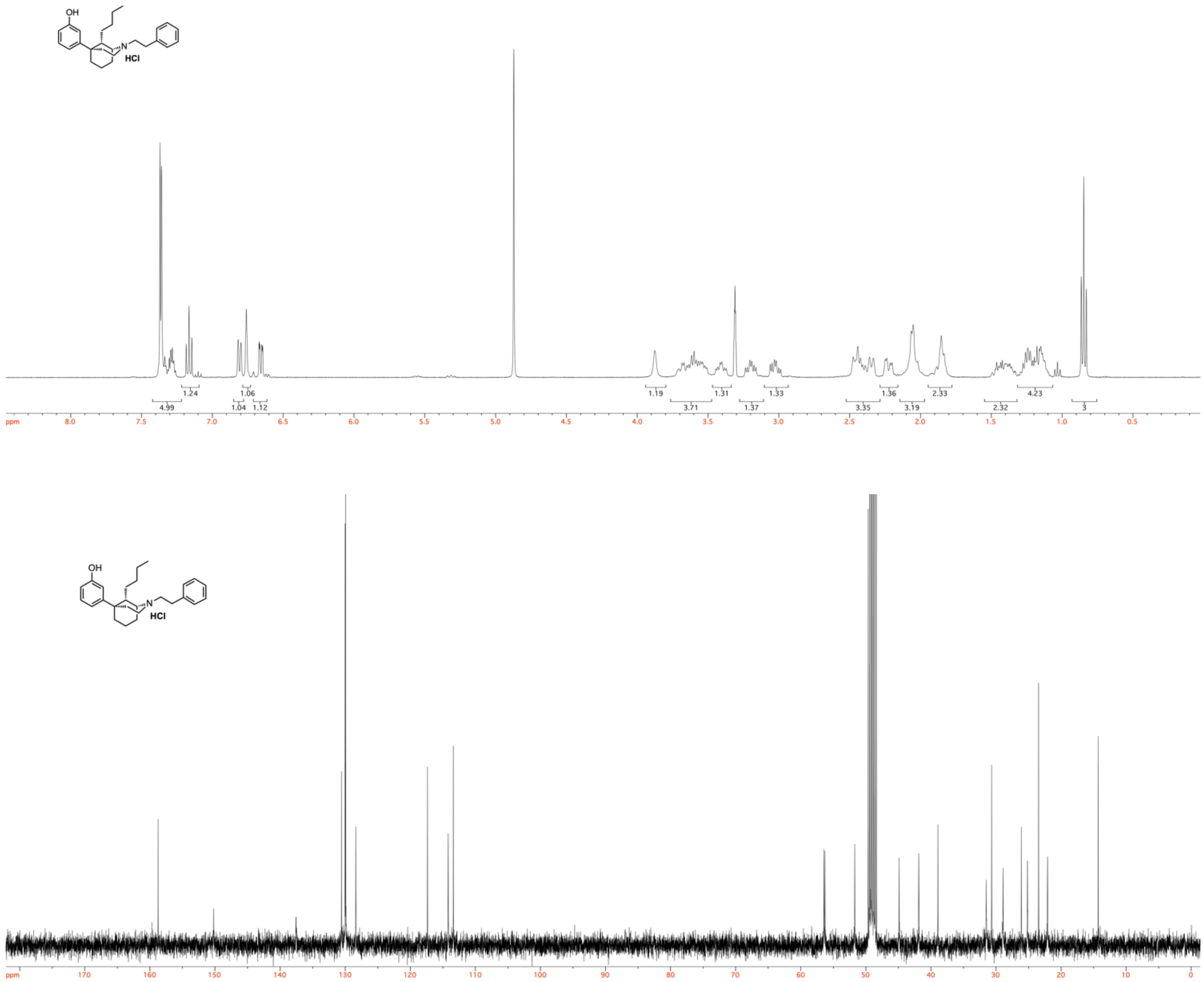

Figure S10: ^{1}H and ^{13}C-NMR spectra of compound **40** (C9*S*)

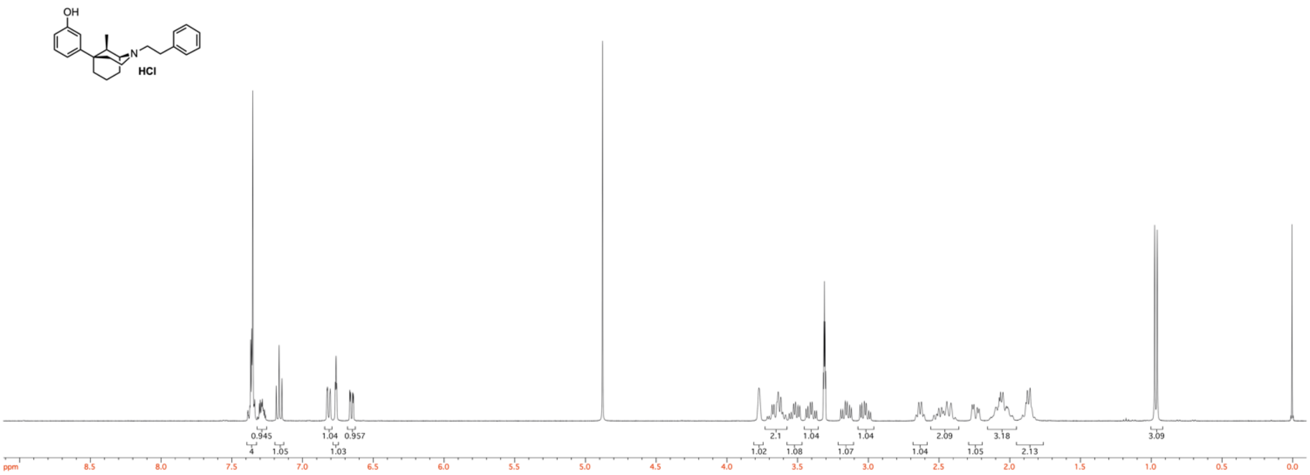

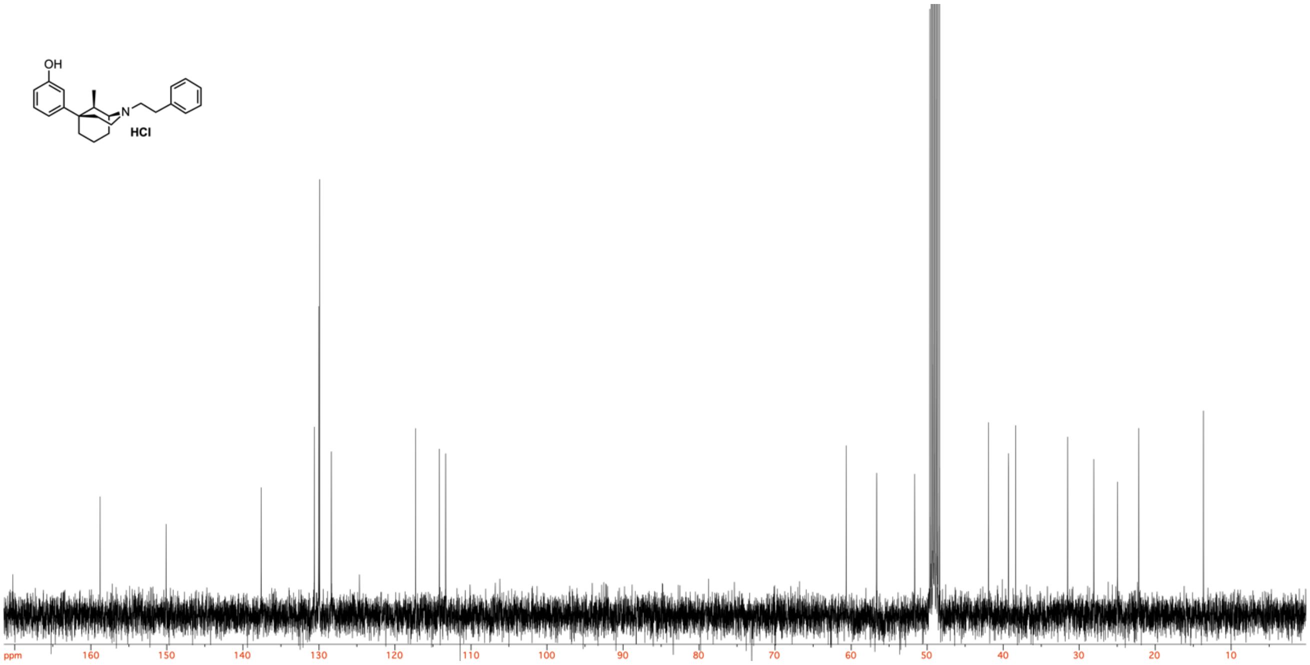

Figure S11: ^{1}H and ^{13}C-NMR spectra of compound **48**

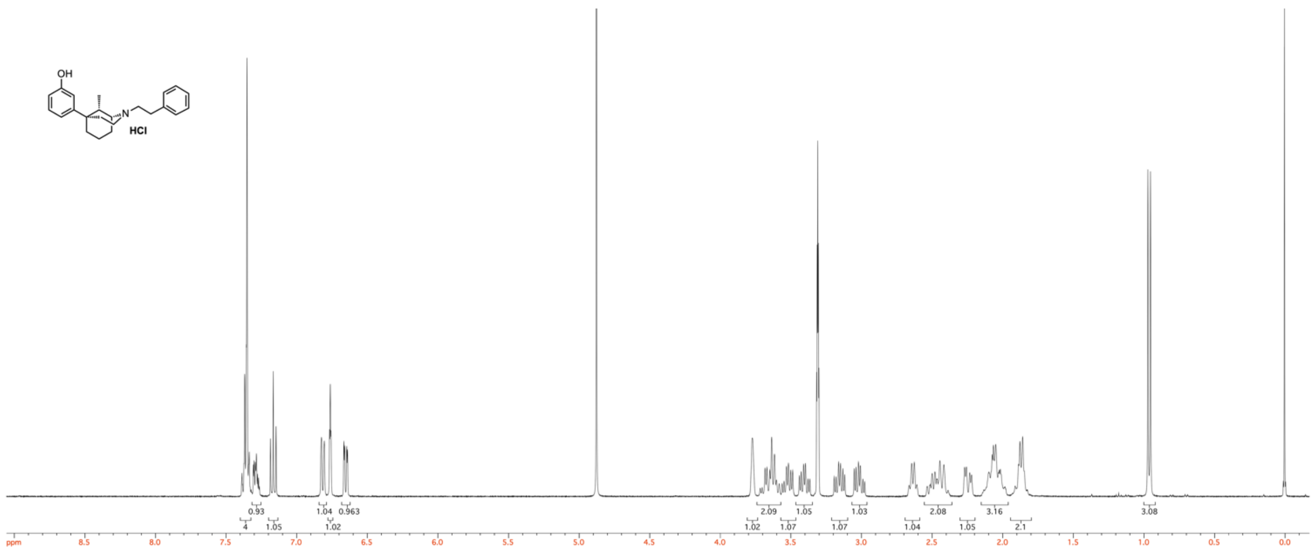

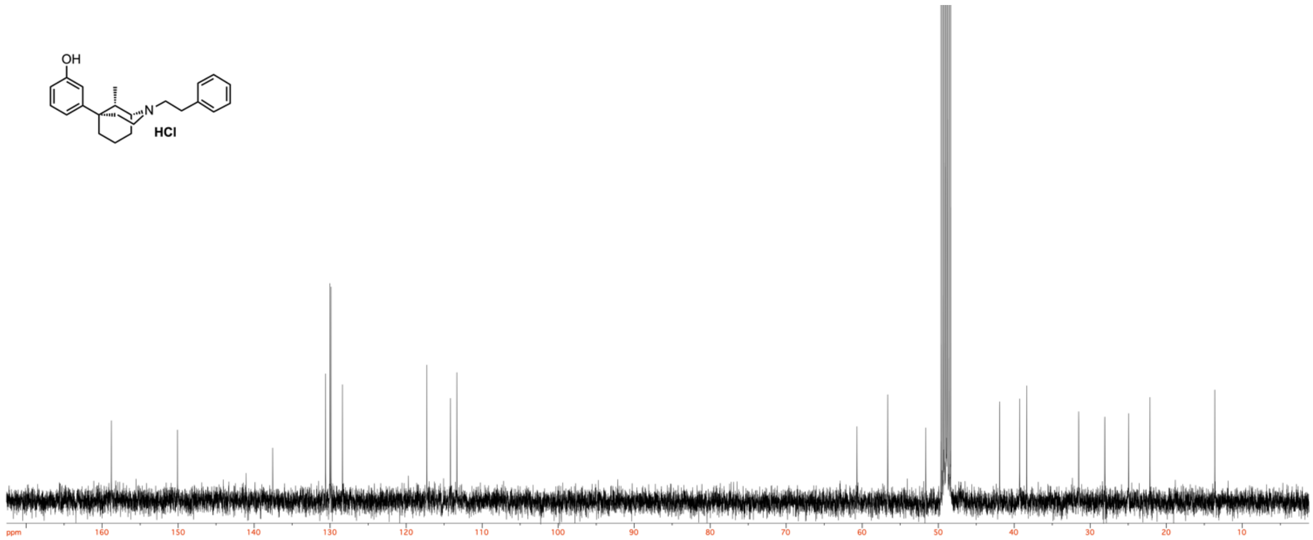

Figure S12: ^{1}H and ^{13}C-NMR spectra of compound **56**
